# Structural Changes and Transport Properties of YBa_2_Cu_3_O_7_ Locally Modified by a He^+^ Focused Ion Beam

**DOI:** 10.1002/advs.77506

**Published:** 2026-09-16

**Authors:** Ross Carter, Robin Hutt, Paul Zimmermann, Ainur Abukaev, Jan Ullmann, Simon Koch, Christoph Schmid, Manfred Burghammer, Reinhold Kleiner, Dieter Koelle, Edward Goldobin, Ivan A. Zaluzhnyy

**Affiliations:** ^1^ Institut für Angewandte Physik Universität Tübingen Tübingen Germany; ^2^ Physikalisches Institut Center for Quantum Science (CQ) and LISA^+^ Universität Tübingen Tübingen Germany; ^3^ European Synchrotron Radiation Facility Grenoble France

**Keywords:** focused ion beam, high‐tc superconductors, josephson junction, nanofocused x‐ray diffraction, phase transitions

## Abstract

Irradiation of a material with ions can cause various defects that can lead to structural phase transitions and the modification of the material's properties. Here we study the irradiation of epitaxially grown thin films of the high‐temperature superconductor YBa_2_Cu_3_O_7_ with 30 keV ${\mathrm{He}}^{+}$ ions which leads to the expansion of the crystal lattice, degradation of superconducting properties, and eventual transition to an insulator. Fabrication of such insulating regions with a focused He‐Ion beam with a spot size of ∼10 nm is a powerful technique for fabrication of superconducting nano‐devices. Using low‐temperature resistivity measurements, diffraction with a nanofocused X‐ray beam and atomic force microscopy, we investigated how the structure and the electric transport properties of YBa_2_Cu_3_O_7_ depend on the irradiation dose in a range 10–100 ions/nm^2^ and on the lateral size of the irradiated area in a range 30–5000 nm.

## Graphical abstract

The structure and the electric transport properties of YBa_2_Cu_3_O_7_ modified with 30 keV ${\mathrm{He}}^{+}$ ions depend on the irradiation dose and lateral size of the irradiated area, as shown by low‐temperature resistivity measurements, diffraction with a nanofocused X‐ray beam and atomic force microscopy. The results are important for fabrication of superconducting devices such as Josephson Junctions.

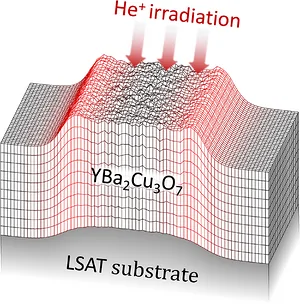

## 1 Introduction

The ability to change the electrical properties of a material on the nanoscale is a crucial technological process in superconducting electronics. The irradiation of superconducting thin films with a focused He‐ion beam (He‐FIB) was established recently as a new nano‐fabrication technique, which was subsequently applied to various superconducting materials [1]. For example, using Nb films one can fine‐cut constriction‐type Josephson junctions (cJJs) and create superconducting quantum interference devices (SQUIDs) based on them [2]. Josephson junctions with a barrier layer (bJJs) formed by He‐FIB irradiation were created based on MgB_2_ films [3, 4, 5], Co‐doped BaFe_2_As_2_ films [6] and NbTiN films [7].

Especially promising is the use of He‐FIB to modify the properties of cuprate superconductors, with ${\mathrm{YBa}}_{2}{\mathrm{Cu}}_{3}O_{7-\delta }$ (YBCO) being a particularly prominent example. He‐FIB was utilised to create artificial pinning nano‐arrays with a lattice spacing as low as 20 nm (matching field of 6T) [8], which allowed for the demonstration of new physical effects, such as the formation of an unusual Bose glass of vortices [9, 10]. Several groups have demonstrated an excellent functionality of bJJs [1, 11, 12, 13, 14] and SQUIDs [12, 15] fabricated by He‐FIB, and investigated THz properties and Shapiro steps for bJJs [16] and bJJ arrays [17]. Recently more advanced devices, for example Josephson diodes, have been demonstrated with record figures of merit [18], as well as constriction‐type Josephson junctions (cJJs) with a constriction width comparable with the He‐FIB spot size ∼10 nm [19].

Despite the substantial use of the He‐FIB in superconducting nano‐electronics, the structural changes caused by the ion irradiation at nanoscale, and their relation to the electronic properties, remain obscure. It has been established that the ${\mathrm{He}}^{+}$ ions create long‐living defects in the YBCO crystal lattice, with a radiation‐dose‐dependent lifetime [20]. If we consider only single lines “drawn” by He‐FIB, it was assumed up to now that lower line‐dose irradiation (≲200 ions/nm) displaces some of the chain O‐atoms, thus changing the effective doping of YBCO and reducing the critical temperature $T_{c}$ [21, 22, 23, 24, 25, 26]. Moderate line‐dose irradiation (∼ 400–700 ions/nm) suppresses $T_{c}$ down to zero. Consequently, since the He‐FIB line has a width of only few nanometres, one can observe Josephson tunneling through such a barrier, i.e., obtain a bJJ. For still higher line‐dose values, the Josephson critical current density decreases and barrier resistance increases exponentially with the dose [12]. For line‐doses ≳1500 ions/nm, the number of defects within the irradiated line becomes so large that the YBCO crystal turns into an amorphous material. This effect can be clearly seen by scanning electron transmission microscopy (STEM) on samples irradiated by ions, electrons and neutrons [8, 12, 19, 27, 28, 29, 30]. In addition to the formation of point defects, the unit cell parameters a, b and c of the orthorhombic YBCO lattice also change on average upon irradiation [25].

Moreover, the structural changes in YBCO seem to depend on the size of the irradiated area [31]. This does not allow us to directly apply the broad range of results obtained on large‐area YBCO films irradiated with an unfocused ion beam [21, 22, 24, 25, 32, 33, 34, 35] to the nanopatterns fabricated by He‐FIB [36, 37]. Therefore, the studies of locally modified YBCO with sufficient spatial resolution are imperative to understand the correlation between crystal structure and superconducting properties.

In this work, we shed light on structural changes of YBCO upon He‐FIB irradiation, focusing on dose‐values below the threshold of amorphisation. We report on the investigation of irradiated areas using different techniques: (a) local spatially resolved X‐ray diffraction (XRD) studies with an X‐ray spot size ∼100 × 100 nm^2^, (b) resistance R vs. temperature T measurements, and (c) atomic force microscopy (AFM) scans. YBCO areas of varying length, ranging from 30 to 5000 nm, were irradiated with doses from 10 to 100 ions/nm^2^. We studied the dependence of both the in‐plane and out‐of‐plane lattice parameters of YBCO on the irradiation dose and irradiation area, correlating the structural changes with the transport measurements performed on the same YBCO chip.

## 2 Results and Discussion

### 2.1 Samples

**FIGURE 1:**
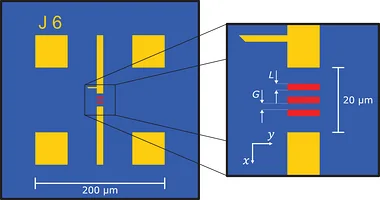
An example of one XRD block. On top of pristine YBCO (blue) there are Au (gold) alignment markers. The irradiation pattern for this XRD block consists of three stripes (red) of the size 10 × 2 $\mu m^{2}$ and spaced by 2 μm. The dose values D=10,20,30 ions/nm^2^ were used for this particular block. The inset shows a magnification of the irradiated area.

We prepared epitaxially‐grown c‐axis oriented YBCO on (100)‐oriented single crystal ${\left({\mathrm{LaAlO}}_{3}\right)}_{3}$ ${\left({\mathrm{Sr}}_{2}{\mathrm{AlTaO}}_{6}\right)}_{7}$ (LSAT) substrates (Figures S1–S4). The 10 × 10 mm^2^ chip was divided into two sections: a transport section, with devices to measure the resistivity of irradiated areas; and an XRD section for the spatially‐resolved structural studies (see Figure S5). The XRD section of the chip initially consisted of one vast YBCO area, connected to ground for charge sink during FIB irradiation, overlaid with Au. This area was then patterned (simultaneously with the transport part, see below) into an array of 200 × 200 $\mu m^{2}$ blocks (Figure 1) by lithographically removing sections of Au, leaving only the alignment marks. Thereafter, using He‐FIB with ion energy 30 keV, each block was irradiated to create a series of stripes (rectangles L×10 μm) of length L interspaced with non‐irradiated (pristine) YBCO spacings of length G. Each subsequent, irradiated stripe had an increasing dose D in the range 10…100 ions/nm^2^. The values of L and G were different between the blocks starting from L=G=500 nm so that one has ten linearly increasing D‐values in the above range within one single block. We also irradiated stripes with L=G=1000,2000,5000 nm, in which case one needs several XRD blocks to cover the whole D‐range specified above, and the D‐values were quasi‐logarithmically spaced from 17…100 ions/nm^2^.

**FIGURE 2:**
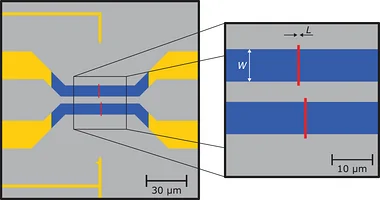
The geometry of microbridges. After lithography and etching down to the LSAT substrate (light grey) we are left with the microbridges consisting of a YBCO‐Au bilayer of width W (here W = 8 μm and length 50 μm connected to the contact pads. In the central part of each microbridge, the Au layer is then removed so that one sees pristine YBCO (blue). These regions are used for irradiation by He‐FIB. The irradiated stripes (red) have a length L (here L = 60 nm) in the transport direction and exceed the bridge width W to avoid shunting through the edges.

The transport section of the chip consists of two identically structured 5 × 5 mm^2^ sub‐chips (see Figure S5). Within each sub‐chip, we lithographically created a set of microbridges, with width W=2 or 8 μm and length 50 or 100 μm, connected to contact pads for 4‐point measurements (Figure 2). We then irradiated rectangular areas across the microbridges using He‐FIB, creating a set of structures with parameters summarized in Table 1.

**TABLE 1:** The parameters of the irradiated areas in the transport part of the chip. W is the width of the microbridge, L is the length of the irradiated rectangle in the transport direction.

W [μm]	L [nm]	D [ions/nm^2^]
2	30	10, 20 …100
8	150	10, 20 …80
8	300	10, 20 …60
8	420	10, 20 …60
8	600	10, 20 …60

### 2.2 Nanofocused X‐Ray Diffraction

We focused X‐rays to the size of approximately 100 × 100 nm^2^ (FWHM) to record the scattering signal exclusively from a small area illuminated by the X‐ray beam. We selected two diffraction peaks of YBCO to measure at each spatial position, predominantly 004 and 104 (Figure S2), to obtain the out‐of‐plane lattice parameter, c, and the in‐plane lattice parameter, a. The specific choice of the reflections was done based on the following criteria: the peak should be above the sample's horizon, it should not overlap with the reflections from LSAT or Au, and it should have enough intensity for effective analysis. It should be noted that for the narrowest stripes, L=500nm, the choice was made to observe the 007 diffraction peak. This is because the position of diffraction peaks at higher angles is more sensitive to minute changes in lattice constant, which allowed us to better resolve the small changes in c found at this L (as seen in Figure 5). This decision comes with the caveat that the same sensitivity to minute changes could potentially bias $D_{dis}$ (introduced later in this section) due to the differences in strain and intensity between different lattice planes.

**FIGURE 3:**
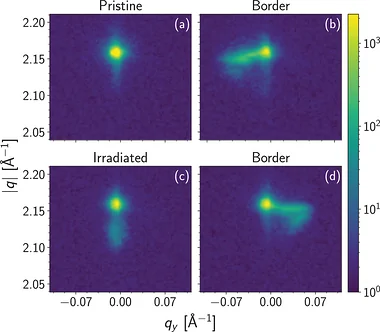
Scattered intensity $I(|q|,q_{y})$ of the 004 diffraction peak of YBCO at different positions across an L=2000 nm stripe which had been irradiated with D=25 ions/nm^2^. (a) An entirely pristine region of YBCO. (b) A transition region from pristine to irradiated YBCO, measured on the spacing‐stripe boundary. (c) An entirely irradiated region. (d) A transition region from irradiated to pristine YBCO, measured on the opposite stripe‐spacing boundary.

**FIGURE 4:**
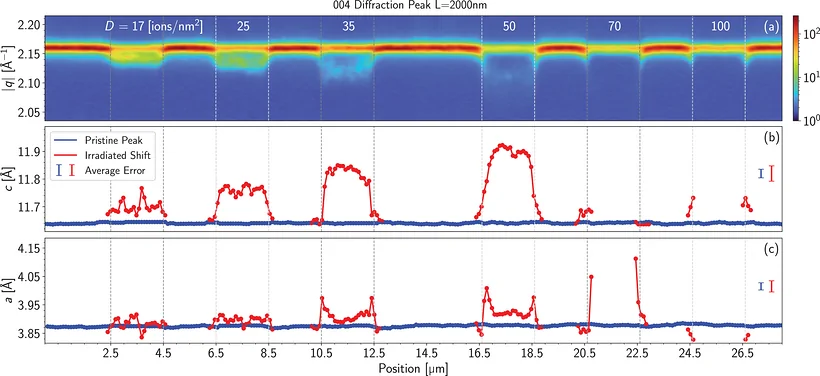
(a) |q|‐profile of the 004 peak of YBCO (integrated over $q_{y}$) at different positions across stripes of L = 2000 nm with increasing dose from left to right. The dashed vertical lines denote the boundaries of irradiated regions. (b,c) Swelling of the (b) out‐of‐plane lattice parameter, c and (c) in‐plane lattice parameter, a across YBCO film with irradiated stripes of length L = 2000 nm.

**FIGURE 5:**
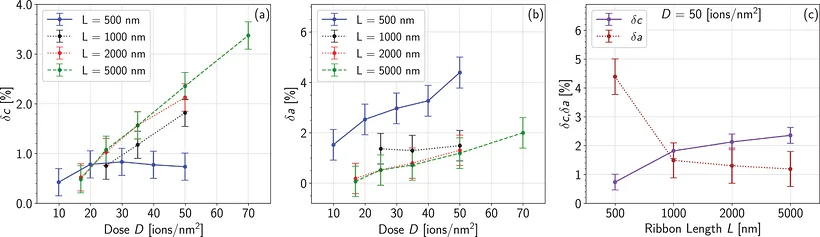
(a,b) Relative change in lattice constants δc (a) and δa (b) for different doses $D\leq D_{dis}^{\left(00l\right)}$, the critical‐disorder dose in the (00l) direction, measured at each L. (c) A comparison of δc and δa for different stripe lengths L, at a constant dose D=50 ions/nm^2^.

For the analysis of the YBCO lattice parameters, we aligned the sample to the Bragg condition by choosing an appropriate orientation of the substrate, determined by the angle ω, and scanned the He‐FIB fabricated stripes with a nanofocused X‐ray beam. We averaged the diffraction patterns measured at the same x‐position across the stripes over y (see Figure S2b). In the same light, the X‐ray beam penetrates the entire depth of the YBCO film, meaning that the diffraction patterns are inherently averaged over that full depth. We are therefore not sensitive to depth‐dependent effects of ${\mathrm{He}}^{+}$ ion irradiation, and can be reasonably certain of consistency when considering which subvolumes are probed during measurements of the 004/007 and 104 Bragg peaks.

Subsequently, we re‐sampled the averaged diffraction patterns from detector coordinates to q‐space using the pygid package [38] and custom, author‐developed scripts. The examples of $I(|q|,q_{y})$ in the selected region of interest (ROI) around the 004 diffraction peak, where $\left|q\right|=\sqrt{q_{x}^{2}+q_{y}^{2}+q_{z}^{2}}$, are shown in Figure 3. Each panel shows how the reflection changes depending on where the X‐ray beam illuminates, relative to an irradiated stripe.

We then employed 2‐dimensional Gaussian fitting on each 004 and 104 diffraction peak to extract their parameters, most importantly its position q=|q|. Where appropriate, e.g. the diffraction pattern in Figure 3c, we treat the pattern as two separate peaks, one pristine peak characterised by a q‐position consistent with pristine YBCO, and one diffuse peak at smaller q values. In these cases, two distinct Gaussian fits were used to obtain the q‐positions of both peaks.

Figure 4a shows the q‐profile of the 004 peak of YBCO for stripes with L = 2000 nm. The fit results are shown in Figure S6. An immediate observation is that the pristine peak remains visible for all D. However, for D>50 ions/nm^2^ its intensity decreases, which is likely related to the partial amorphisation of the YBCO crystal lattice. We attribute the persistence of the weaker, but still relatively sharp, pristine peak observable for D>50 ions/nm^2^ to nanoscale grains (fragments) of the YBCO crystal lattice that retain their own internal structure, even inside the irradiated stripes. This is consistent with previous research [31], where it was observed that some small fraction of pristine YBCO remained in irradiated areas and stripes when irradiating with an FIB. This effect is possibly owing to the local nature of irradiation when using a FIB, as the irradiated material cannot freely expand due to contact with neighbouring (non‐irradiated) regions, so the pristine crystal structure of YBCO is stabilised close to the substrate. The pristine peak persists up to the highest irradiation dose D=100 ions/nm^2^, and only disappears at a much higher amorphisation dose $D_{c}$. In the STEM studies [8, 12, 19], it was observed that when $D>D_{c}$, the crystal amorphises in the irradiated region; here we observe similar effects, albeit less pronounced due to the smaller doses used.

The appearance of a diffuse peak at slightly smaller q‐values than those for the pristine peak indicates that some fraction of the YBCO lattice expands and loses its perfect crystal order due to the formation of structural defects. This peak undergoes a greater change in q with increasing irradiation dose D, reaching a maximum change at a critical value, $D_{dis}$. At doses $D>D_{dis}$, the disorder in the YBCO crystal lattice is so high that the diffuse peak becomes indistinguishable from the background, and the signal‐to‐noise is so small that reliably fitting the peak is not possible. We therefore define the critical‐disorder dose, $D_{dis}$, as the maximum dose for which we still observe a diffuse peak; notably this dose is much smaller than the previously described amorphisation dose, $D_{c}$. We are confident that $D_{dis}$ is related to the decrease of intensity of the diffuse peak, as demonstrated in Figure S4.

One exception to this behaviour is the boundary between pristine and irradiated regions, see Figure 3b,d. Here, the behaviour of the diffuse peak more closely resembles the previously discussed behaviour of the pristine peak, whereby it remains even at the highest irradiation dose D=100 ions/nm^2^. This is likely because the neighbouring pristine region anchors the irradiated YBCO, which prevents complete disorder and thus the erosion of the diffuse Bragg peak. The appearance of the diffuse peak on the side of the main reflex (as opposed to directly below it) indicates that the lattice planes are tilted at the transition region between the pristine and irradiated YBCO, as will be discussed in Section 2.3. Even with this tilt, the diffuse peak remains near the Ewald sphere, as shown in Figure S3, which allows us to extract its position and intensity with high accuracy.

The q‐position of the 004 peak allows us to directly calculate the lattice constant $c=4\cdot 2\pi /q_{004}$, presented in Figure 4b. The lattice in this example expands by approximately 0.2 Å in the c‐direction at D=50 ions/nm^2^, in agreement with previous works with unfocused [25, 32] and focused [31] ion beams. Subsequently, we investigated the 104 peak utilising the same methods as above (see Figure S7). Using the q‐position of the 104 peak in conjunction with the previously calculated out‐of‐plane lattice constant c, we determined the in‐plane lattice constant

(1) $$a=\frac{1}{\sqrt{{\left(\frac{q_{104}}{2\pi }\right)}^{2}-{\left(\frac{4}{c}\right)}^{2}}},$$

which is shown in Figure 4c. The value of a increases by approximately 0.1 Å at the same dose. Such an increase of the in‐plane lattice parameter for small irradiated areas is drastically different from the only small changes of the average in‐plane lattice parameter observed for unfocused ion irradiation [31]. We explain this unexpected expansion of the in‐plane lattice parameter by the bending discussed in section 2.3. This difference in the behaviour of the in‐plane lattice parameter for focused and unfocused ion irradiation indicates that the strain at the boundary between the irradiated and pristine YBCO can alter the structural transition in YBCO and modify its properties in dependence of the size and shape of the irradiated area.

The same analysis was also employed for stripes of L=500,1000 and 5000 nm (see Figures S8–S13). Figure 5 summarises the observed changes in the lattice parameters as a function of dose D and length L (see also Figures S14–S16). Here, δc=Δc/c and δa=Δa/a represent the relative change in lattice constants c and a respectively, averaged across each irradiated stripe (along x‐axis in Figure S2b, excluding stripe‐spacing boundaries). At L=500 nm, δa is approximately 4.4% at D=50 ions/nm^2^, significantly greater than observed in unfocused (broad beam) irradiation studies [31] or when changing the oxygen deficiency in ${\mathrm{YBa}}_{2}{\mathrm{Cu}}_{3}O_{7-\delta }$ [39, 40]. Therefore, at high doses, it is not possible to resolve the crystallographic transition from the orthorhombic to the tetragonal phase. However, based on previous publications [31], we believe that this transition still occurs for the YBCO samples presented in this study. Between L=500 and 1000 nm there is a steep decrease in δa, before a more gradual decline as L increases; juxtaposed to this, δc observes a sharp increase in the same L range, before increasing more gradually at larger L.

Another key finding is that the value of the critical‐disorder dose, $D_{dis}$, obeys two key trends. First, it tends to be slightly higher for larger values of L; second, it is consistently smaller for the 004 Bragg peak than for the 104 Bragg peak (Table 2), with the exception of the largest stripe L=5000nm, where they were equal. The latter trend suggests that the defects induced by the He‐FIB irradiation destroy the crystal order in the out‐of‐plane direction (c‐direction) first.

**TABLE 2:** Summary of each investigated diffraction peak and the corresponding critical‐disorder dose $D_{dis}$.

Peak	L [nm]	$D_{dis}$ [ions/nm^2^]
007	500	50
004	1000	50
004	2000	50
004	5000	70
104	500	60
104	1000	70
104	2000	70
104	5000	70

One caveat to the methods presented in this chapter is that we require the out‐of‐plane lattice constant c to calculate the in‐plane lattice constant a, meaning that we can only reliably measure a up to the critical‐disorder dose in the (00l) direction, $D_{dis}^{\left(00l\right)}$, despite the critical‐disorder dose being larger in the (h0l) direction. Furthermore, the YBCO film twinning [41] along ⟨110⟩ directions did not allow us to distinguish the a and b orthorhombic lattice parameters. Instead, the lattice constant derived from the 104 Bragg peaks represent an average in‐plane lattice parameter. For this reason, we cannot resolve the change in lattice constant that would be indicative of an orthorhombic to tetragonal phase change even at lower doses.

### 2.3 Atomic Force Microscopy

**FIGURE 6:**
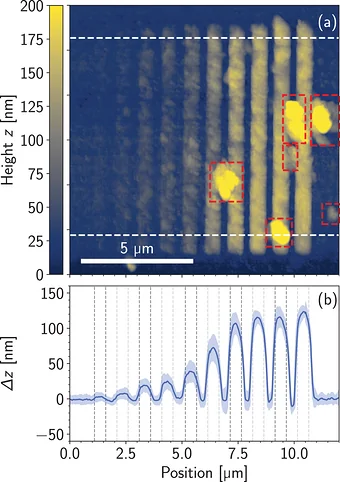
(a) AFM image (height z) measured across the 10 stripes of length L=500 nm irradiated with doses D=10,20,…,100 ions/nm^2^. The dashed, white lines represent the limits of the average profile in (b), the red boxes represent masked regions used to eliminate artefacts. (b) Change of the height Δz relative to the pristine YBCO region. The shaded area represents the roughness of the film. The vertical dash lines indicate the borders of irradiated stripes.

Figure 6a shows the height z of ten irradiated stripes measured by the AFM. Here, we observe an increase in height of our film in irradiated regions up to Δz≈130 nm for the dose D≈80 ions/nm^2^, after which Δz saturates (Figure 6b). This *bending* up is far larger than the *swelling* — predicted changes due to an increase in c, which would have been on the order of a few nm. This strongly indicates up‐bending of both the YBCO film and LSAT substrate, probably caused by the formation of He bubbles deep inside LSAT, given the fact the ${\mathrm{He}}^{+}$ ions mostly penetrate through the YBCO film and are stopped in the substrate [31].

The amount of bending appears to be the same for all L≥500 nm for the doses D<50 ions/nm^2^. For higher doses (D≥50 ions/nm^2^), the change in height Δz due to bending is maximal at L=500 nm, and is slightly less pronounced for both smaller and larger L (see Figure S18).

At the dose of D=50 ions/nm^2^, bending was not observed at L = 30 nm, the narrowest stripe that was measured with AFM (Figure S18). This indicates that if L is small enough then the restrictive boundaries of non‐irradiated YBCO+LSAT prevent any such bending. Additionally, this correlates with STEM images taken on the same L = 30 nm stripe, where no significant change in the stripe was observed [30].

### 2.4 Transport Measurements

**FIGURE 7:**
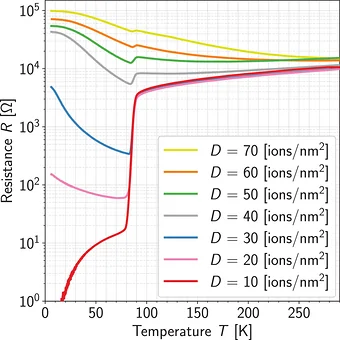
R(T) curves ($I_{b}=1\;\mu A$) of YBCO microbridges irradiated with different values of D in the area L×W= 30 nm × 2 μm.

Figure 7 shows the R(T) curves measured on fabricated microbridges (Figure 2). One can see that below $T_{c}$ the resistance of irradiated areas grows quite rapidly over several orders of magnitude with D (which is correlated with structural changes, see, for example, Figure 4a) at a given T. This is compatible with a transport via variable‐range hopping [42]. For low values of D, e.g., D=30 ions/nm^2^, the temperature plays an important role in the transport properties, such that R changes one order of magnitude between T=10 and 80 K, however, the effect of T is much weaker for large D.

We have also performed similar R(T) measurements for bridges housing irradiated areas of different sizes, see Table 1. Since below $T_{c}$ the pristine banks of YBCO are superconducting, the measured resistance originates entirely from the irradiated areas. The R(T) dependences show similar behaviour; however, the resistances proved to be much larger than expected from the scaling with L/W. Many R(T) curves for D>40 ions/nm^2^ could not be measured reliably due to saturation limits of the setup.

**FIGURE 8:**
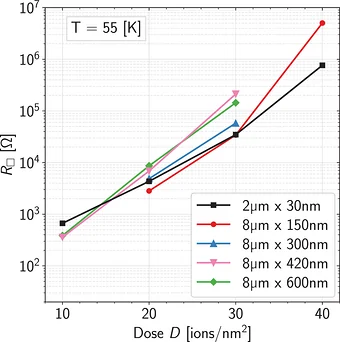
The dependence of the sheet resistance $R_{□}\left(D\right)$ of the irradiated stripes for the low D range, where a comparison is possible. Different curves correspond to different irradiated area size.

To visualise the non‐linear growth of resistance with L/W, we have extracted the resistance per square (sheet resistance) $R_{□}=R\cdot W/L$ at T=55 K (chosen to maximise the number of data points), shown in Figure 8. In the usual case when R∝L/W, the dependencies $R_{□}\left(D\right)$ should all overlap and go along one line. As shown in Figure 8, $R_{□}$ also increases strongly (more than one order of magnitude) with L/W at fixed D, which is not the case for a usual conductor. This, however, is expected if the swelling or bending depends on the size of the irradiated area. Note that the dependence corresponding to W = 2 μm has a less steep increase. This is probably related to the fact that the size effect depends not only on L, but also on W, and this is the only bridge with W = 2 μm. This shows that $R_{□}$ depends not only on the size of irradiated area, but also on its shape, which might be related to border regions between the pristine and irradiated YBCO, that are bent and experience the highest strain and therefore might also exhibit poorer transport properties.

### 2.5 Discussion

**FIGURE 9:**
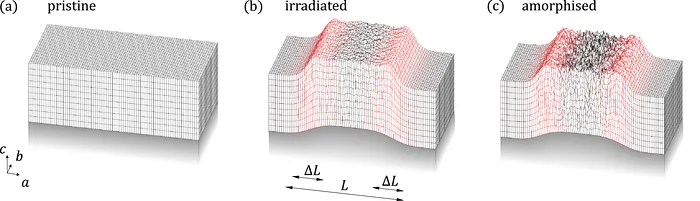
Visual representation of YBCO lattice expansion and substrate bending. (a) Pristine YBCO with unchanged lattice constants (grid on top) and pristine LSAT substrate (shaded region below). (b) A stripe with length L of YBCO irradiated with $D\leq D_{c}$. Lattice expands in c and a directions by a small amount. The film also experiences bending at the border ΔL due to He ion bubbles. Areas experiencing the greatest strain are coloured red. (c) A stripe of amorphised YBCO with $D>D_{c}$. Too many defects have been introduced to the lattice causing overwhelming strain. There is no longer a consistent structure and there is significant bending.

Results derived from nanofocused X‐ray diffraction suggest that amorphisation is a product of an increasing strain in, and the ultimate “structural collapse” of, the lattice. This is due to the swelling lattice constants and bending in the LSAT substrate, which in turn are due to the large quantities of defects introduced at higher D. Figure 9 shows an exaggerated representation of how the YBCO lattice changes with increasing dose, from pristine to amorphised.

The critical‐disorder limit, $D_{dis}$, is correlated to the length L of irradiated stripes. At small L the lattice is anchored closely by its neighbouring pristine regions. These constraints cause an increased strain on the lattice due to lattice expansion (swelling) and greater film bending, making $D_{dis}$ smaller. Conversely, at large L, the film is less constrained and the lattice has more leeway to expand before the strain becomes too great, so $D_{dis}$ increases.

We hypothesise that this bending allows the in‐plane lattice parameters a to expand by up to ∼4.4% (Figure 5b). From the AFM images of the large irradiated areas (5 × 10 $\mu m^{2}$), we see that the whole irradiated area is elevated by Δz (see Figure S17). This means that the strongest curvature of the crystal lattice occurs at the border ΔL between the irradiated and pristine YBCO, while in the centre of the irradiated area the lattice planes are again more or less straight. Assuming that there is a limit to the lattice plane bending, i.e. there is the maximum possible height change Δz which the YBCO film can accommodate, we see that for large irradiated areas the lateral expansion δa cannot exceed a certain limit. Otherwise, the limited bending of the lattice plane cannot compensate the overall increase of the lateral size of the irradiated area, which is proportional to ∝L·δa. This argument is supported by the dependence of δa on L shown in Figure 5c.

The expansion of the lattice parameter c also causes strain predominantly at the border ΔL between the irradiated and pristine stripes due to anchoring by the pristine area. However, the value of this strain does not depend on the size of the irradiated area, since the expansion of the lattice in the vertical direction is unrestricted outside of ΔL, which remains roughly the same regardless of stripe size. Therefore, we can assume that for large sizes of the irradiated area L, the lattice can expand in the vertical direction more freely. This is also supported by Figure 5c, in which the value δc increases with the size L.

For the very small irradiated areas (a few tens of nm, L≲ΔL), we expect that there will be only very small changes in the lattice parameters because of a small irradiated area trapped inside the pristine material which does not allow any significant deformations.

## 3 Conclusions

In this work, we used nanofocused X‐ray diffraction to measure the change of the YBCO lattice parameters caused by He‐FIB as a function of irradiation dose and irradiation area. Surprisingly, both the c and a lattice constants increase with D. The change of the in‐plane lattice parameter a is larger than what was observed earlier on the samples irradiated by unfocused ${\mathrm{He}}^{+}$ beam [31]. This indicates that the structural changes caused by local irradiation, e.g. by He‐FIB, differ from the changes caused by broad‐beam irradiation.

At the critical‐disorder dose, $D_{dis}$, one observes a decrease of intensity for both the 004 and 104 peaks corresponding to a sudden loss of order in the large volume fraction of the crystal. We clearly observe a size‐effect: $D_{dis}$ increases with the length L of the irradiated stripe embedded into pristine crystal. Moreover, this size effect is evident when considering the relative change in the lattice constants, δc and δa. As L increases, we note that δc also increases, whereas δa decreases. We also observe a marginally different $D_{dis}$ for the order in a and c directions.

Using AFM, we observe changes in the film's height on a scale approximately one order of magnitude larger than the lattice expansion; which we interpreted as a bending of the substrate. This bending agrees with the opposite trends that is seen in δc and δa when the size of the irradiated area L is changed (see Figure 5c). The bending in the irradiated stripes with a large length L allows for more expansion in c, while keeping the increase in a moderate, due to the strongest curvature occurring exclusively at the stripe boundaries. By similar logic, the stripes with small L have a larger perimeter‐to‐area ratio, thus δc is smaller, and δa larger.

Transport measurements show a superconductor‐insulator transition with resistance quickly increasing with the dose D. In contrast to X‐ray diffraction, we do not see any abrupt changes in resistivity when the dose exceeds $D_{dis}$. However, the size effect is also visible: the areas with larger L exhibit up to one order of magnitude larger sheet resistance $R_{□}$ than those with small L. The dose‐dependent correlations between structural and transport effects provide quantitative guidelines for selecting irradiation conditions that achieve controlled electrical isolation while preserving the structural integrity of the surrounding superconducting material, which is important for reliable fabrication of high‐density superconducting circuits.

Our studies demonstrate that by irradiating narrow stripes one can create a resistor with resistance value tunable by dose and temperature over several orders of magnitude. Making the stripes longer (in transport direction) makes not only the resistance, but also the resistivity larger due to a size‐effect related to the swelling and bending of the irradiated region embedded into fixed pristine YBCO crystal. These findings enable a better understanding of how ${\mathrm{He}}^{+}$ irradiation parameters can be tuned to modify the properties of superconducting YBCO films. Our work provides a foundation for the rational design of FIB‐fabricated JJs, superconducting weak links, single‐photon detectors, and other high‐temperature superconducting electronic circuits.

## 4 Experimental Section

### 4.1 Sample Fabrication

The samples were fabricated by pulsed laser deposition (PLD) of epitaxially‐grown c‐axis oriented YBCO on (100)‐oriented single crystal ${\left({\mathrm{LaAlO}}_{3}\right)}_{3}$ ${\left({\mathrm{Sr}}_{2}{\mathrm{AlTaO}}_{6}\right)}_{7}$ (LSAT) substrates. The thickness of YBCO was 100 nm, which is larger than the typical 30–50 nm used for fabricating Josephson junctions and circuits, due to the need for a stronger X‐ray scattering signal. The sharp diffraction peaks from YBCO on θ−2θ scans with a full width at half maximum (FWHM) of $6\cdot 10^{-3}$ Å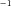 (Figures S1 and S2) indicate that the films can be considered a high‐quality single crystal in the vertical direction. In the in‐plane direction, it is expected to exhibit ⟨110⟩ twinning, typical for orthorhombic YBCO [31, 41]. The critical temperature for the pristine YBCO film was measured at $T_{c}∼88$ K.

### 4.2 Irradiation With ${\mathrm{He}}^{+}$ Ions

The microbridges and stripes on the YBCO film were fabricated by a Zeiss Orion NanoFab helium ion microscope using the 30 keV ${\mathrm{He}}^{+}$ ion beam.

When irradiating areas, the dose is naturally measured in ions/nm^2^. It is useful to have a relation between the 1D and 2D dose values that have the same effect, e.g., amorphisation, on the sample. This relation depends on the FIB spot size σ (assuming it has a Gaussian shape with the standard deviation σ, which is typically 2–5 nm). The value of σ is supposed to be much larger than the pitch size (pixel size of a helium ion microscope positioning system, which can be made as small as 0.25 nm). In this simplified model the relation between $D^{1D}$ given in ions/nm and $D^{2D}$ given in ions/nm^2^ having the same effect on the sample is

$$D^{1D}=\sqrt{2\pi }\sigma \cdot D^{2D}$$

For example, if the amorphisation of lines starts at $D_{c}^{1D}\approx 1800$ ions/nm then the amorphisation of areas will start at $D_{c}^{1D}\approx 180$ ions/nm^2^, assuming σ≈4 nm.

### 4.3 Synchrotron Experiment

The nanofocused X‐ray diffraction experiment [43] was carried out at the beamline ID‐13 of the European Synchrotron radiation facility (ESRF). The X‐ray beam with energy E=15 keV was focused to the size of approximately 100 × 100 nm^2^ (FWHM) using multilayer Laue lenses [44, 45]. The stripes on the YBCO chip fabricated by He‐FIB were raster scanned on a rectangular grid with resolution 100×1000 nm^2^ (vert. × hor.), owing to an increased footprint of the X‐ray beam in horizontal direction when the sample is aligned for a Bragg condition. Thus, the spatial resolution of approximately 100 nm was achieved in the direction perpendicular to the stripes (Figure S2). The large EigerX 4M detector placed 260mm behind the sample was used to record the diffraction patterns from the film.

### 4.4 Atomic Force Microscopy

The AFM measurements were performed with the Nanowizard‐II system from JPK–Bruker.

### 4.5 Transport Measurement

The electrical transport measurements were performed at the University of Tübingen after the XRD experiment at the ESRF. For the transport measurements we used microbridges fabricated on the same chip that was used for the XRD experiment. While cooling from T≈300 K (and warming up again) in a liquid He dewar, each microbridge was biased by a constant probe current of $I_{b}$ = 1 μA; the voltage across each microbridge (between bonding pads) was measured in 4‐point‐configuration, thus determining the resistance. During each cooling cycle, four microbridges were measured simultaneously. Note that for $T<T_{c}\approx 88$ K the pristine (non‐irradiated) YBCO film is superconducting; therefore, all measured resistance corresponds to the resistance of irradiated areas. However, at $T>T_{c}$, the non‐superconducting pristine YBCO (as well as the Au covered leads) also contribute to the total resistance shown in Figure 7 (few kΩ for YBCO, and few Ω for Au).

## Author Contributions

R.K., D.K., E.G. and I.A.Z. conceptualised the work; R.H., S.K., C.S. prepared the samples; R.C., R.H., P.Z., J.U. M.B. and I.A.Z. performed measurements; R.C., R.H., A.A., E.G. and I.A.Z. analysed the data; R.C., R.H. and E.G. wrote the manuscript with contribution from all authors.

## Conflicts of Interest

The authors declare no conflicts of interest.

## Supporting information


**Supporting File**: advs77506‐sup‐0001‐SuppMat.pdf.

## Data Availability

The data collected during an experiment at the ID13 beamline of European Synchrotron Radiation Source ESRF (Grenoble, France) are available at https://doi.esrf.fr/10.15151/ESRF‐ES‐1691831423 after an embargo period [46].
