# Supplementary material for: Structural Changes and Transport Properties of YBa2Cu3O7 Locally Modified by a He+ Focused Ion Beam
Source: Adv Sci (Weinh). 2026 Sep 16:e77506. Online ahead of print. doi: 10.1002/advs.77506 (PMC13583111; doi:10.1002/advs.77506)
Supplement: Supplementary file 1 [file ADVS-9999-e77506-s001.pdf]

Supplemental materials to Structural Changes and Transport Properties of $YBa_2Cu_3O_7$ Locally Modified by a He^+ Focused Ion Beam

Ross Carter,[1] Robin Hutt,[2] Paul Zimmermann,[1] Ainur Abukaev,[1]
Jan Ullmann,[2] Simon Koch,[2] Christoph Schmid,[2] Manfred Burghammer,[3]
Reinhold Kleiner,[2] Dieter Koelle,[2] Edward Goldobin,[2] and Ivan A. Zaluzhnyy[1]

[1]*Institut für Angewandte Physik, Universität Tübingen,*
Auf der Morgenstelle 10, 72076 Tübingen, Germany
[2]*Physikalisches Institut, Center for Quantum Science (CQ) and LISA$^+$,*
Universität Tübingen, Auf der Morgenstelle 14, 72076 Tübingen, Germany
[3]*European Synchrotron Radiation Facility,*
71 Avenue des Martyrs, 38000 Grenoble, France

Pre-characterization of the YBCO film on LSAT substrate

Prior the structural investigations with a nanofocused X-ray beam, the YBCO/LSAT chip with Au contacts was characterized using the laboratory diffractometer (GE-3303TT, Cu-$K_{\alpha 1}$ radiation). The measured $\theta - 2\theta$ scan with sharp $00l$ reflections from YBCO is shown in Figure S1. The rocking scan (ω-scan) on the pristine region of the YBCO/LSAT chip was done before the spatially-resolved X-ray diffraction studies (Figure S2(a)). The maximum intensity projection recorded by the detector during the rocking scan is shown in Figure S2(c).

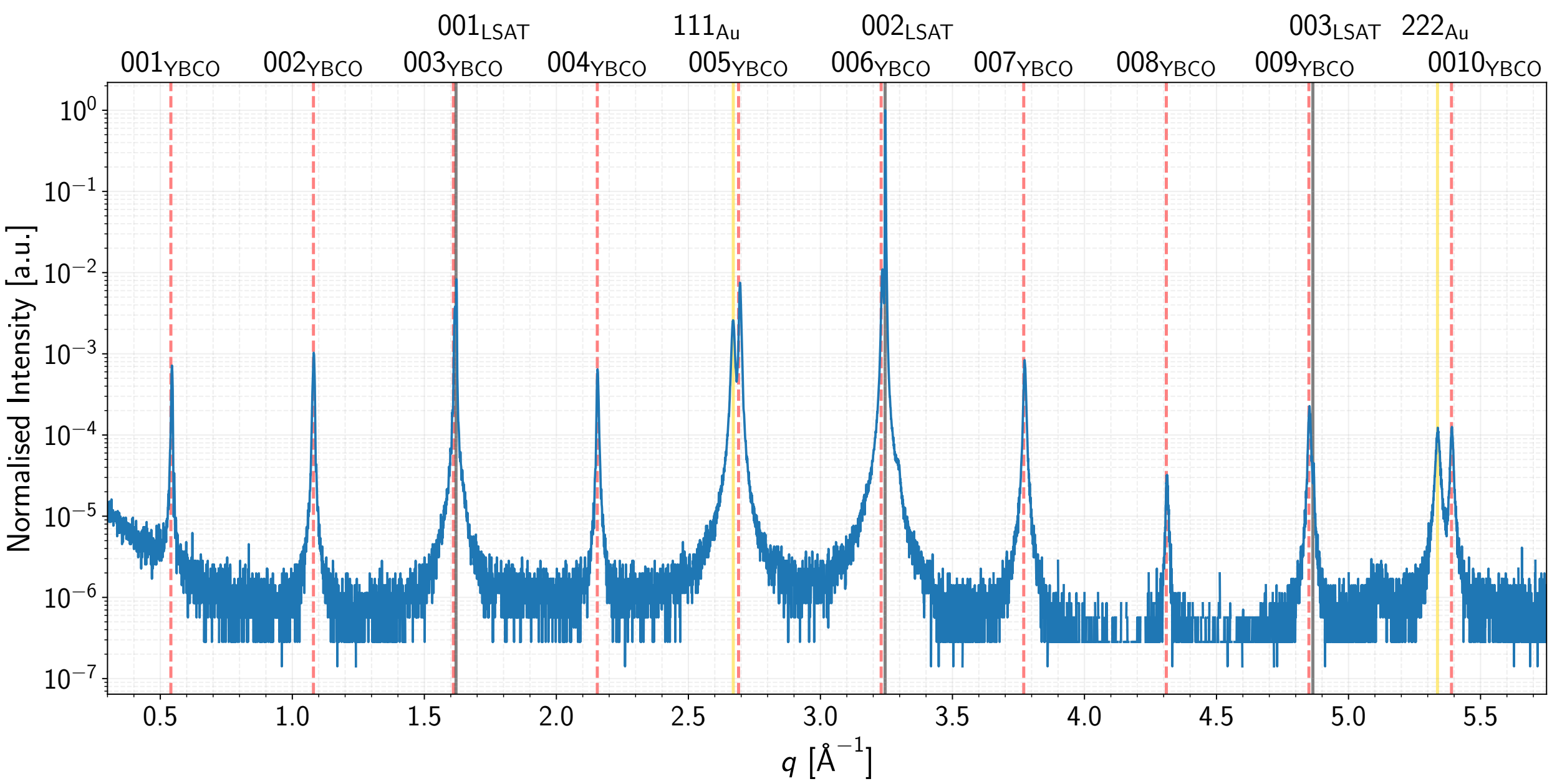

FIG. S1. $\theta - 2\theta$ scan of the YBCO/LSAT chip with Au contacts. Red dash lines mark $00l$ diffraction peaks of YBCO (the extracted lattice parameter c = 11.65 Å), solid gray lines - $00l$ diffraction peaks of LSAT (the extracted lattice parameter a = 3.87 Å), solid yellow lines - 111 and 222 peaks of Au (the extracted lattice parameter a = 4.08 Å). The diffracted intensity is normalized to the maximum value (006 reflection of YBCO).

Geometry of the Nanofocussed X-ray Diffraction Experiment

The X-ray diffraction experiments were performed in reflection geometry in the direction of x in Figure S2(b) (i.e. with the irradiated stripes parallel to the direction of the X-ray beam). The reason why the scanning direction has such importance arises from the bending of the lattice planes described in Section 2.3 of the main text. This point is illustrated by Figure S3, which shows the Ewald sphere (cross-section of the reciprocal space measured by the detector) and a diffraction peak (in this case, 007 reflection), represented as a sphere. If the irradiated stripes were perpendicular to the X-ray beam, as shown in Figure S3(b,c), the tilting of the lattice planes would effectively change the incidence angle ω, and the Bragg reflection would shift across the Ewald sphere. In this case, even for small tilting angles, the Bragg peak would move very fast from the diffraction condition and the measurement of the diffraction signal would not be possible without adjustment of the sample's orientation. However, in this experiment, the irradiated stripes were parallel to the X-ray beam, as shown in Figure S3(d,e). ln this case, the Bragg peak would move along the Ewald sphere while always staying in the diffraction condition. In the diffraction patterns, this would look like shift of the scattering signal to left and right, as shown in Figure 3(b,d) of the main text.

In addition to the raster scans with spatial resolution, we measured rocking curves around the 007 Bragg peak at the centre of large irradiated areas of $5\,\mu m \times 5\,\mu m$ for different doses (see Figure S4). From these measurements, we see that the diffraction pattern consists of a pristine 007 peak at the original position and a diffuse Bragg peak just below it (which corresponds to a larger lattice spacing). Thus, we did not observe any tilt of the lattice planes for large stripes. For the large doses, the diffuse peak becomes less intense, but it does not move further down from the pristine reflection. These rocking curves assure that the definition of the critical-disorder limit, D_{dis}, is related to the decrease of intensity of the diffuse peak, and not to its movement out of scattering condition.

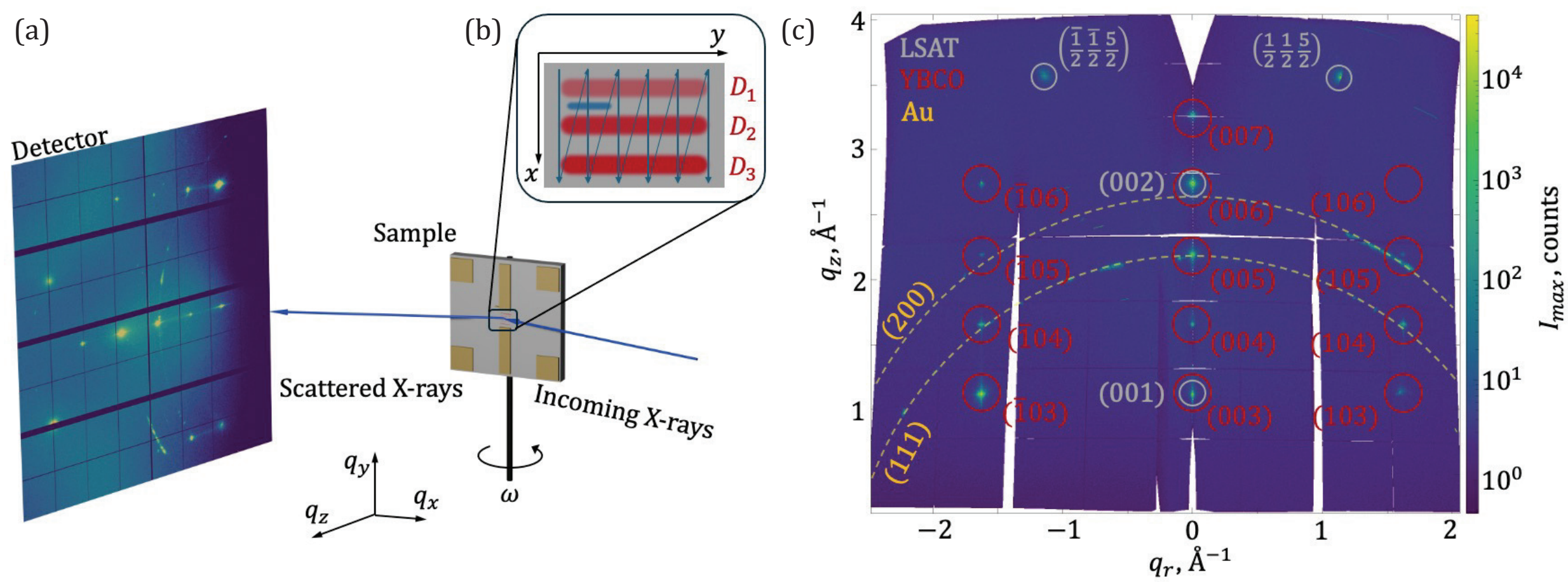

FIG. S2. (a) Geometry of the spatially-resolved X-ray diffraction experiment. For clarity, only a single block with He-FIB pattern surrounded by Au marks is shown. The coordinate system (q_x, q_y, q_z) is chosen with respect to the sample's surface. (b) Scheme of the raster scan of the stripes with doses D_1, D_2, D_3, ... with the X-ray beam (not to scale). The elongated blue spot represents the footprint of the X-ray beam. The diffraction patterns collected for the same position along the stripe (the same value of x-coordinate) were averaged for the better statistics. (c) Maximum intensity projection $I_{\max}(q_r, q_z)$ ($q_r = \sqrt{q_x^2 + q_y^2}$) recorded by the detector during the rocking scan of the YBCO chip on the pristine region (with Au contacts). YBCO peaks are labeled with red, LSAT - with gray, and the position of the Scherrer rings from Au - with yellow. Note that the 001 and 002 peaks from LSAT substrate overlap with the 003 and 006 peaks from YBCO. The intensity of the diffraction pattern (in counts) is represented by color, white regions correspond to the detector gaps and the missing wedge.

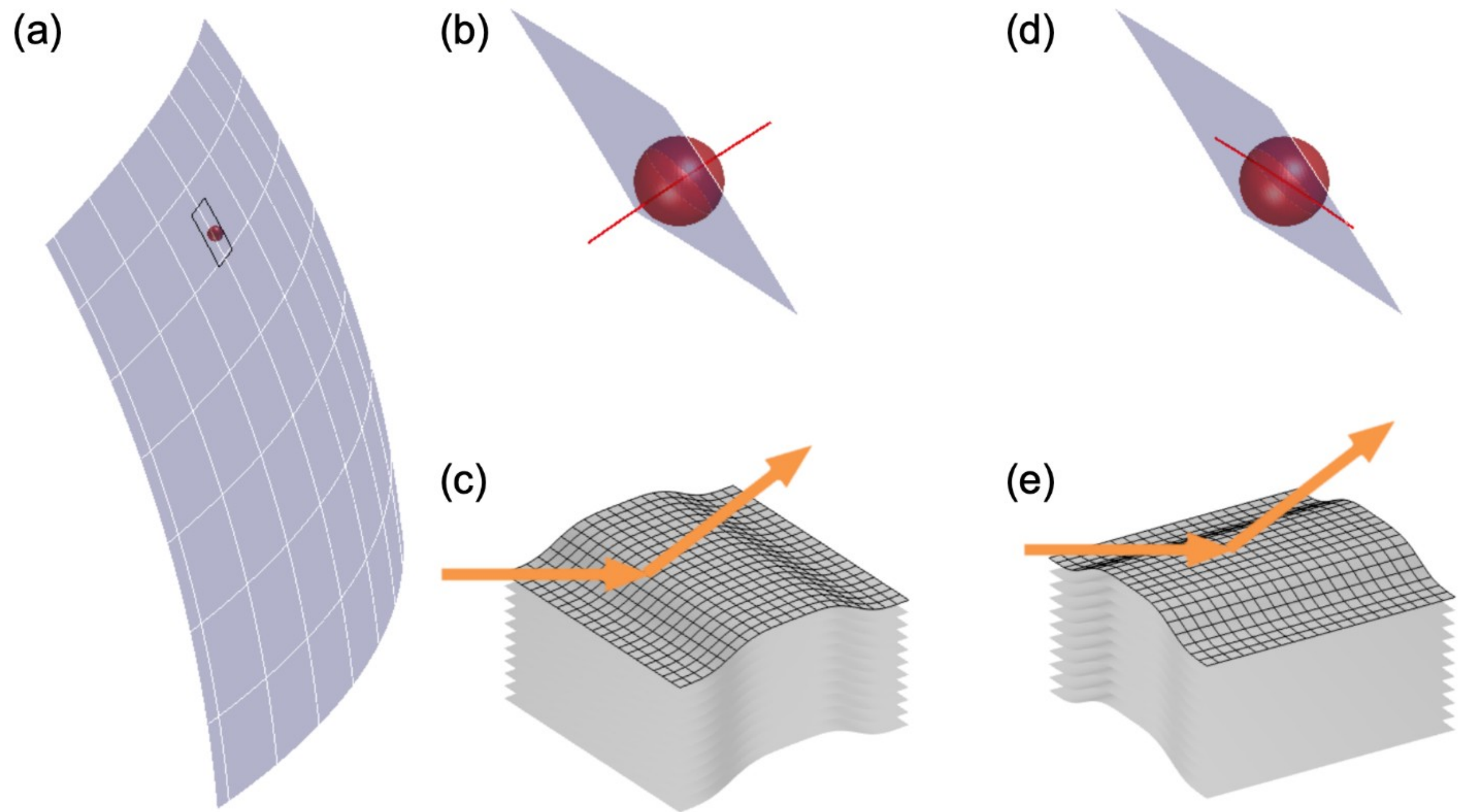

FIG. S3. (a) Ewald sphere (corresponding to the full detector), 007 Bragg peak represented as a sphere with radius 0.05 $\mathrm{\AA}^{-1}$, and the ROI around the Bragg peak shown in black. (b,d) Shift of the Bragg peak due to tilting of the lattice planes in two directions. (c,e) Corresponding tilting of the lattice planes when the X-ray beam (orange) is perpendicular and parallel to the irradiated stripes.

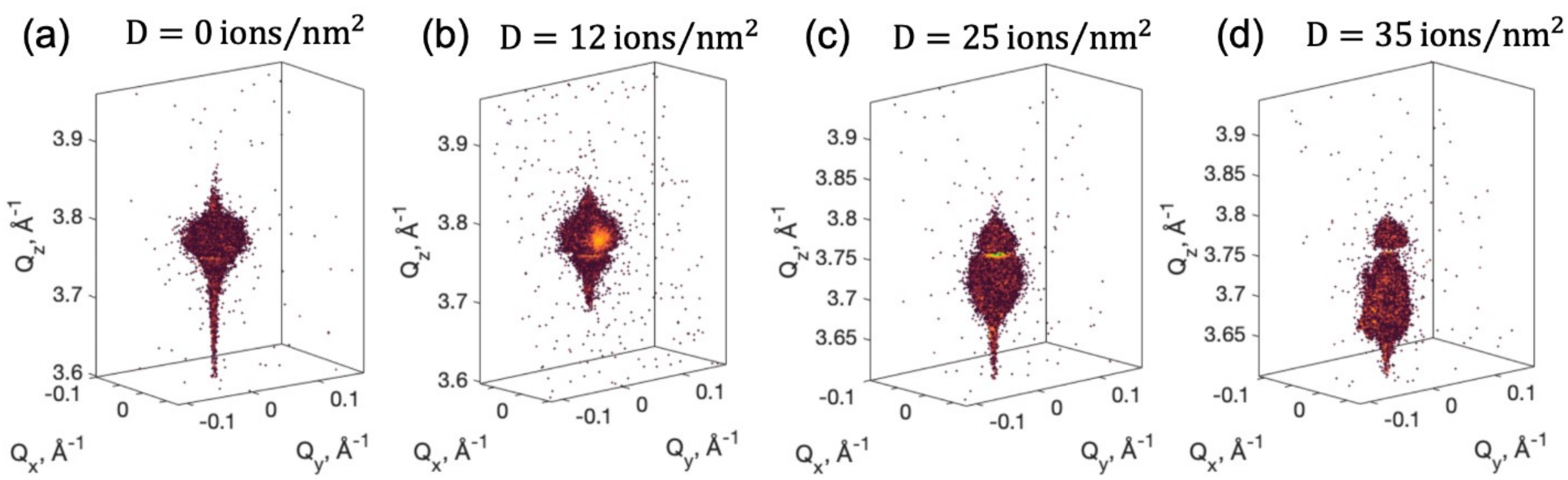

FIG. S4. Examples of the 3D distribution of the scattered intensity around the 007 reflection of YBCO measured in the centre of large areas (5 µm × 5 µm) irradiated with different doses. One can see the remaining pristine peak at about $q = 3.78$ Å^{-1} and a diffuse peak just below. The horizontal gap in 3D distribution arises from the detector gap.

Layout of the YBCO chip

For this experiment two equal sample chips were fabricated. On each chip of size $10 \times 10\,\mathrm{mm}^2$ a $9 \times 9\,\mathrm{mm}^2$ centred square of 100 nm thick of YBCO got fabricated on top of the LSAT substrate. The YBCO film got covered by 20 nm of Au. By using two lithography etching steps on one hand all Au except of the remaining Au (golden in Figure S5) structures got removed on the other hand all YBCO got etched away where plain LSAT is exposed (light gray in Figure S5). That implies in particular that under every Au structure YBCO is present.

The left half of the chip - the transport half - is structured in two equivalent sub-chips ($5 \times 5\,\mathrm{mm}^2$). Each sub-chip consists of a vertical common-ground-conductor, which Au layer is interrupted in the middle to enable 4-point measurements. The individual set of microbridges are connected horizontally to this common-ground-conductor. On the right side of the common-ground-conductor three sets of six microbridges are attached. These microbridges are $50 \times 8\,\mu\mathrm{m}^2$ in size and grouped in two, separated by $5\,\mu\mathrm{m}$ (Figure 2 of the main text) to be irradiated within one field of view ($25 \times 25\,\mu\mathrm{m}^2$). On the left side a set of eight microbridges and a set of ten blue connect to the common-ground-conductor. The set of eight is composed of the same microbridges as the six-microbridge sets while the ten microbridges set splits off to two subsets of five microbridges each. Since the size of those microbridges is $100 \times 2\,\mu\mathrm{m}^2$ each subset of five microbridges fits into the irradiation field of view of size $25 \times 25\,\mu\mathrm{m}^2$.

To enable irradiation of the pristine YBCO the Au on top of each microbridge got removed over a length of $70 - 80\,\mu\mathrm{m}$ during fabrication. Each microbridges end which is not connected to the common-ground-conductor is connected to the microbridges bondpad. The covering Au of the bondpad is split in two to enable 4-point measurement (as the common-ground-conductor).

The right half of the chip - XRD half - homes the 132 XRD blocks. On top of the YBCO layer ($4.5 \times 9\,\mathrm{mm}^2$) these are arranged in a array of twelve lines and eleven columns labelled from A1 to L12. Each XRD block is ($200 \times 200\,\mu\mathrm{m}^2$) in size and the array spacing is $400\,\mu\mathrm{m}$ both horizontally and vertically. Since the array's vertical dimension is just 4.8 mm, and the array is centred to the middle of the YBCO rectangle above and beneath the XRD block array, there is enough pristine YBCO for performing pre-characterization as shown in the

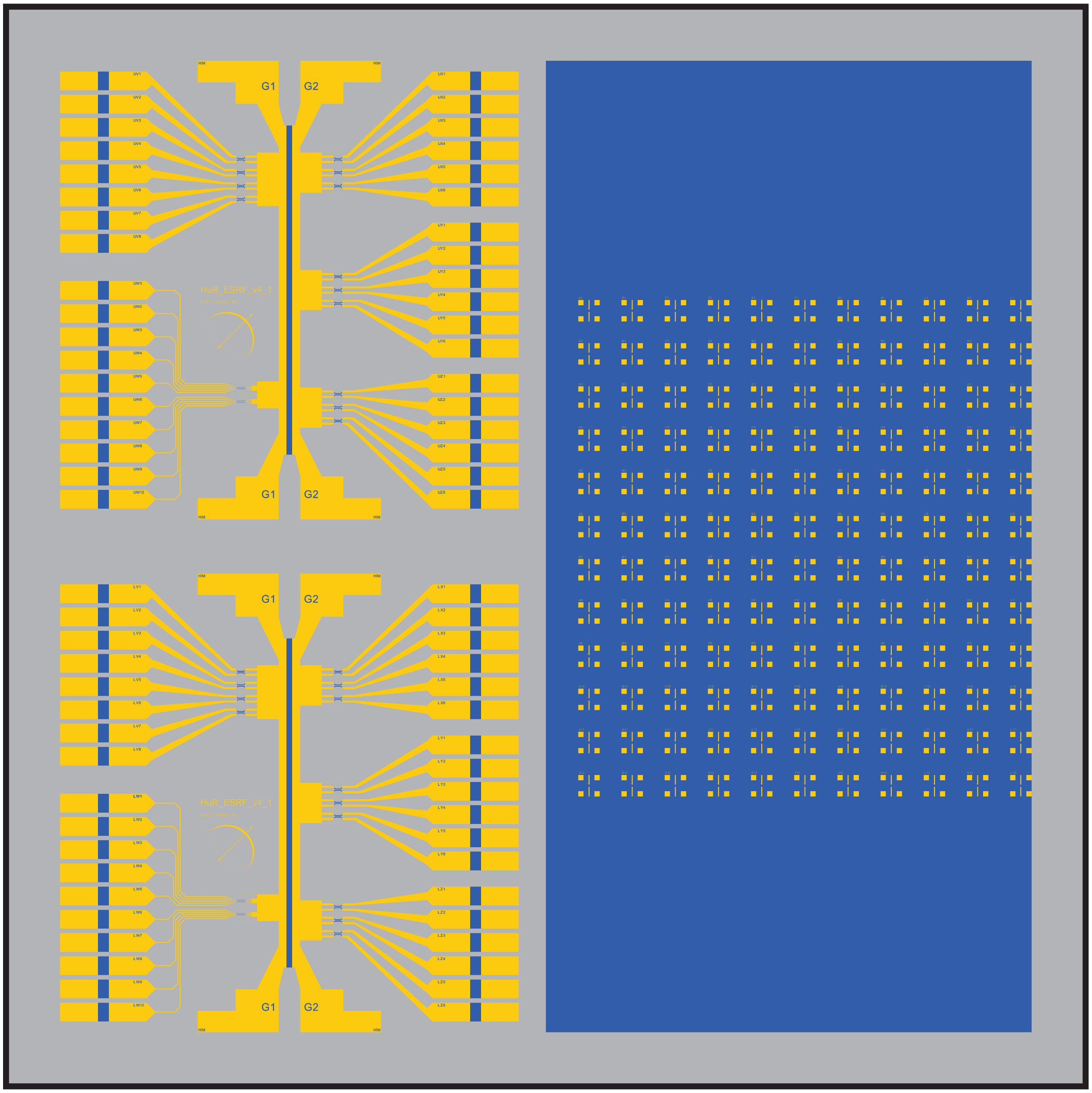

FIG. S5. Chip Layout $10 \times 10\,\text{mm}^2$: On top of the substrate LSAT (light gray) a 100 nm thick layer of YBCO (blue) is fabricated by PLD and covered by 20 nm Au (gold). The left half of the chip -the transport half- is structured in two equivalent sub-chips ($5 \times 5\,\text{mm}^2$), carrying 34 microbridges each connected to the vertical common-ground-conductor. XRD half on the right consists of a large YBCO area with 132 XRD blocks structured in Au to help alignment both for irradiation and XRD investigation.

previous section.

As shown in Figure 1 of the main text, next to the block's identifier four Au squares mark the corners of the XRD block. They are needed to as markers especially for navigating on the chip while irradiation and while aligning the sample during the XRD experiment. A $10\,\mu$m wide vertical Au strip represents a guideline to the (to be) irradiated area. In the centre of the Au strip which is the centre of the XRD block the Au is interrupted for $20\,\mu$m where the irradiation takes place.

Both close to the microbridges and close to the irradiation area in the XRD block focal markers are integrated in the layout. They form a sharp Au tip where the microscope operator can refocus the He-beam directly before irradiation. For the XRD block the focal marker is $\approx 28\,\mu$m to the upper left of the area to be irradiated's centre and horizontally connected to the upper half of the guideline marker. The microbridge's focal marker is $33\,\mu$m above and beneath the microbridges half length.

Additional figures for the main text

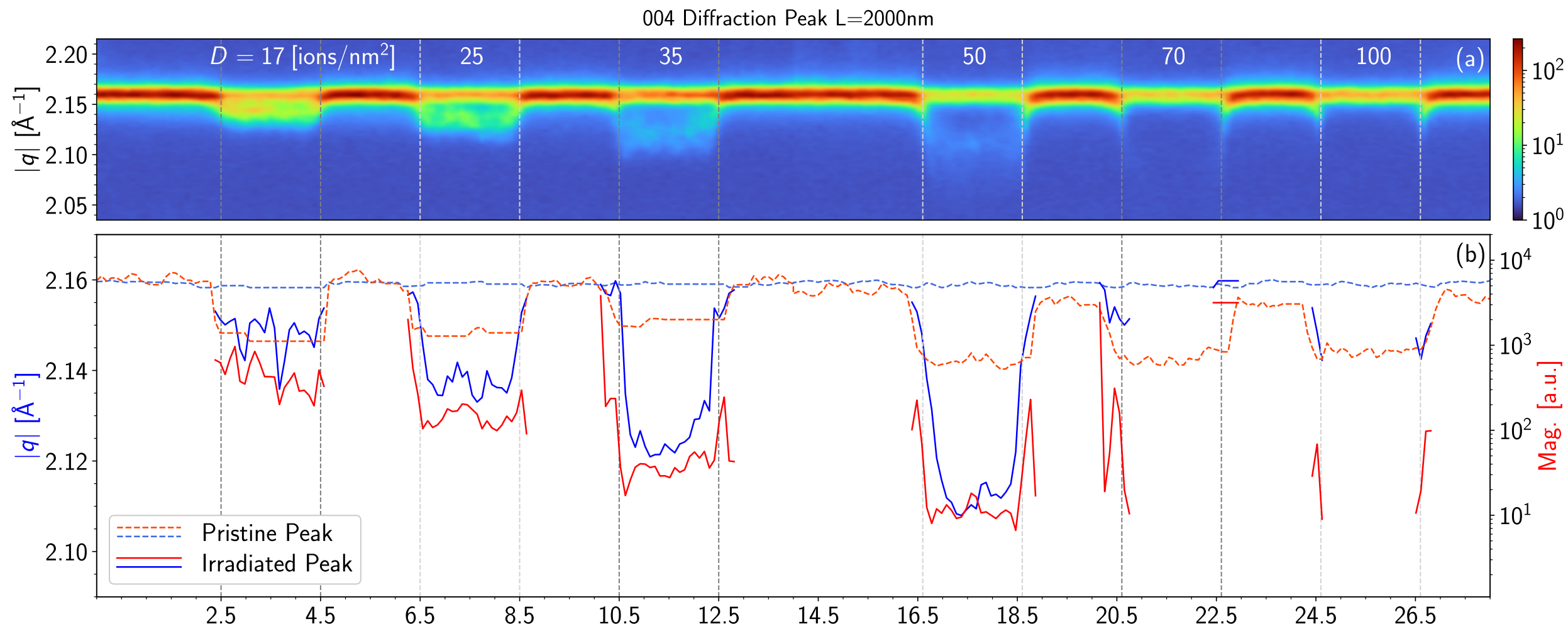

FIG. S6. (a) $|q|$-profile of the 004 peak of YBCO (integrated over q_y) at different positions across stripes of $L = 2000\,\mathrm{nm}$ with increasing dose from left to right. The dashed vertical lines denote the boundaries of irradiated regions. (b) $|q|$-positions (blue) and magnitude (red) of the Gaussian fits of the pristine (dashed lines) and diffuse (solid lines) peaks.

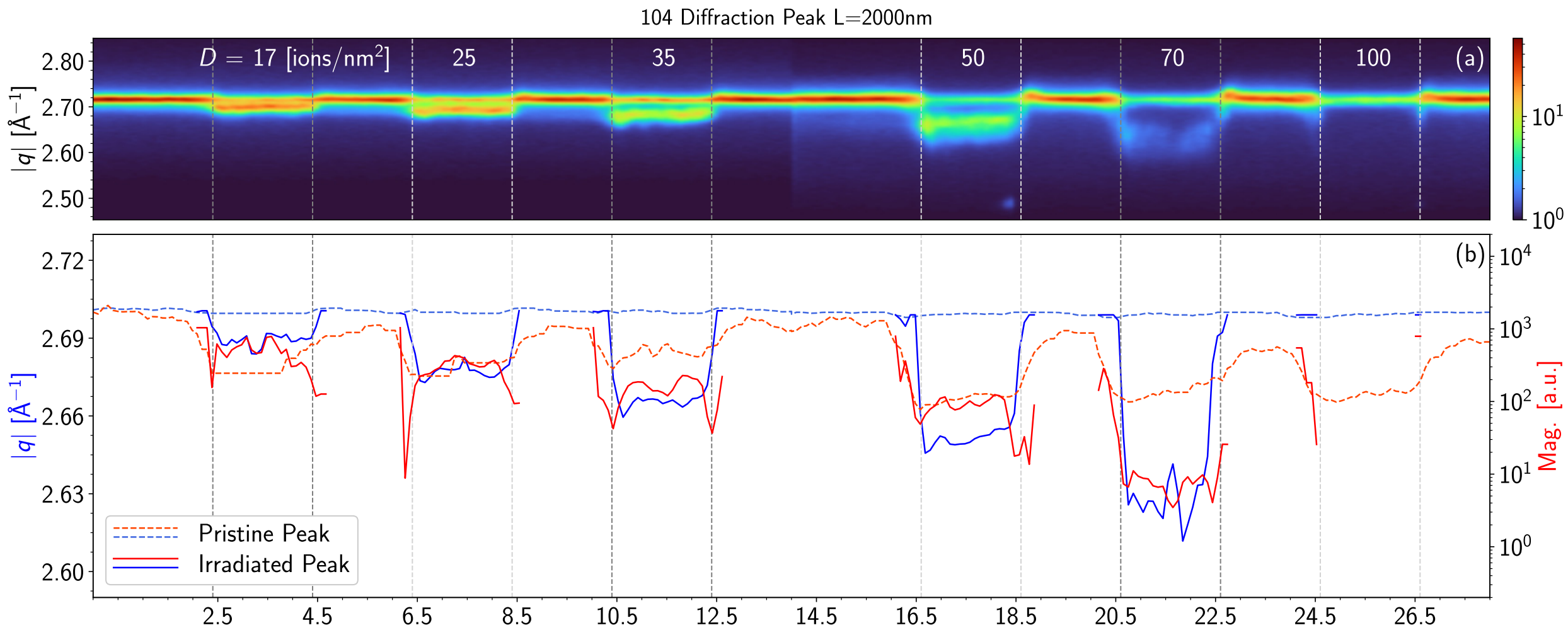

FIG. S7. (a) $|q|$-profile of the 104 peak of YBCO (integrated over q_y) at different positions across stripes of $L = 2000\,\mathrm{nm}$ with increasing dose from left to right. The dashed vertical lines denote the boundaries of irradiated regions. (b) $|q|$-positions (blue) and magnitude (red) of the Gaussian fits of the pristine (dashed lines) and diffuse (solid lines) peaks.

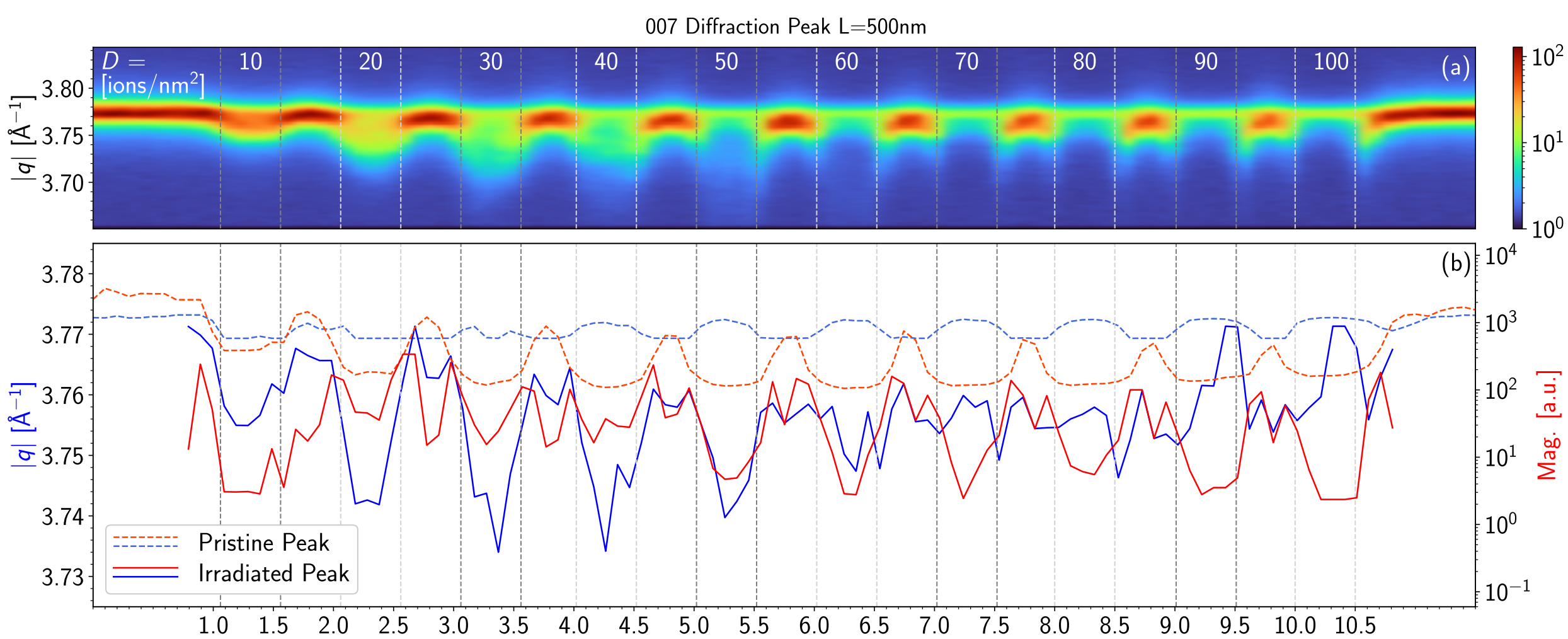

FIG. S8. (a) $|q|$-profile of the 007 peak of YBCO (integrated over q_y) at different positions across stripes of $L = 500\,\mathrm{nm}$ with increasing dose from left to right. The dashed vertical lines denote the boundaries of irradiated regions. (b) $|q|$-positions (blue) and magnitude (red) of the Gaussian fits of the pristine (dashed lines) and diffuse (solid lines) peaks.

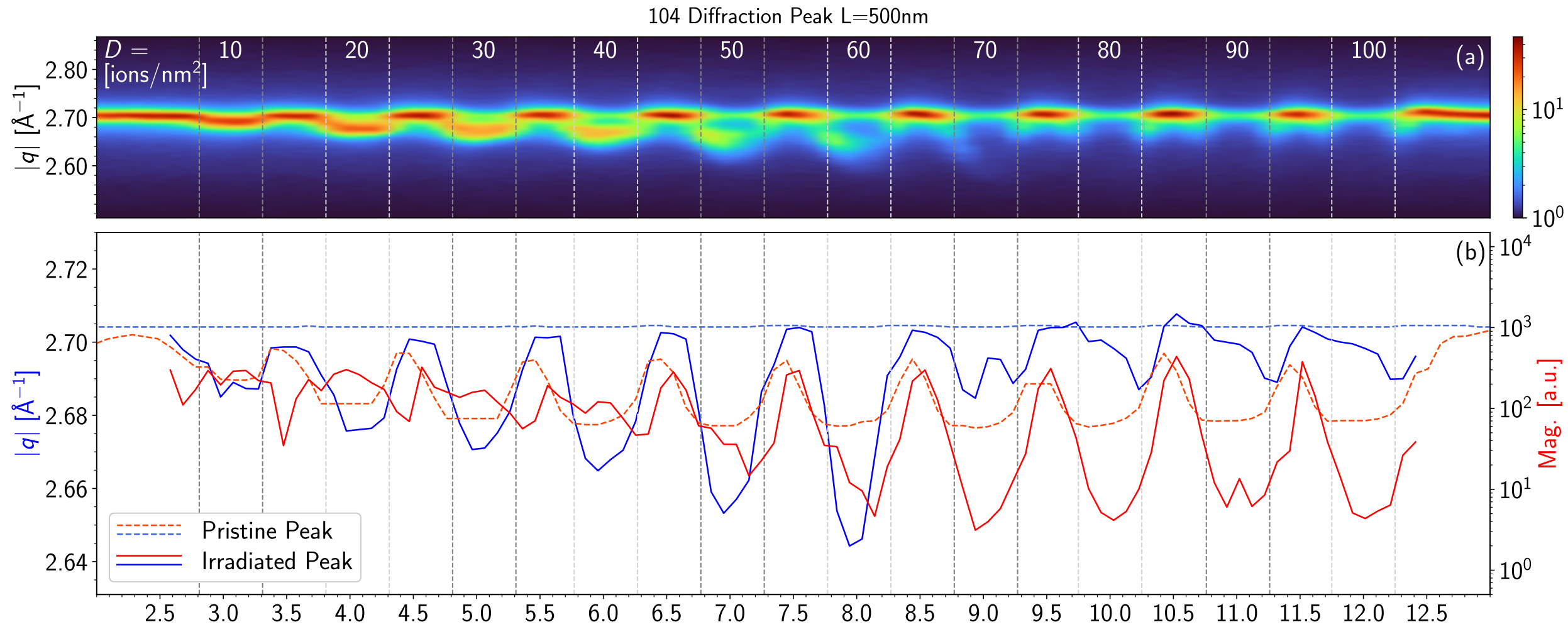

FIG. S9. (a) $|q|$-profile of the 104 peak of YBCO (integrated over q_y) at different positions across stripes of $L = 500\,\mathrm{nm}$ with increasing dose from left to right. The dashed vertical lines denote the boundaries of irradiated regions. (b) $|q|$-positions (blue) and magnitude (red) of the Gaussian fits of the pristine (dashed lines) and diffuse (solid lines) peaks.

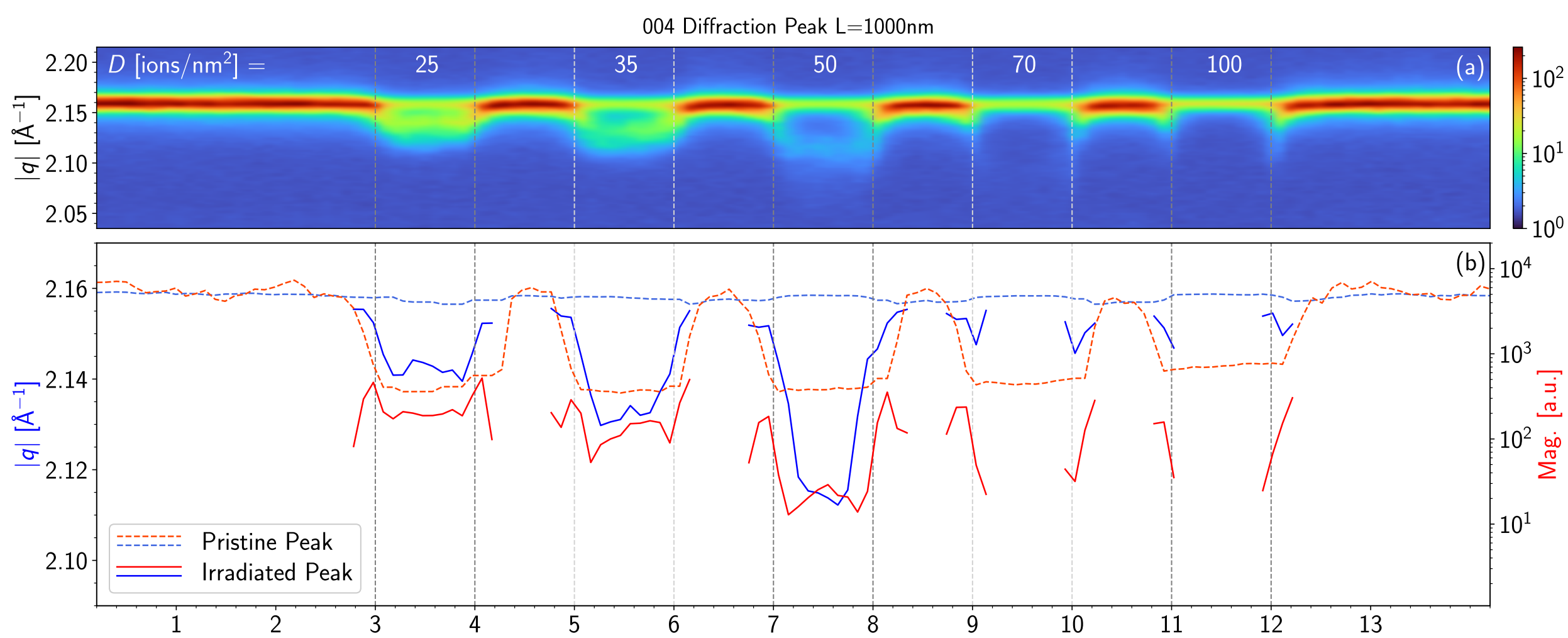

FIG. S10. (a) $|q|$-profile of the 004 peak of YBCO (integrated over q_y) at different positions across stripes of $L = 1000\,\mathrm{nm}$ with increasing dose from left to right. The dashed vertical lines denote the boundaries of irradiated regions. (b) $|q|$-positions (blue) and magnitude (red) of the Gaussian fits of the pristine (dashed lines) and diffuse (solid lines) peaks.

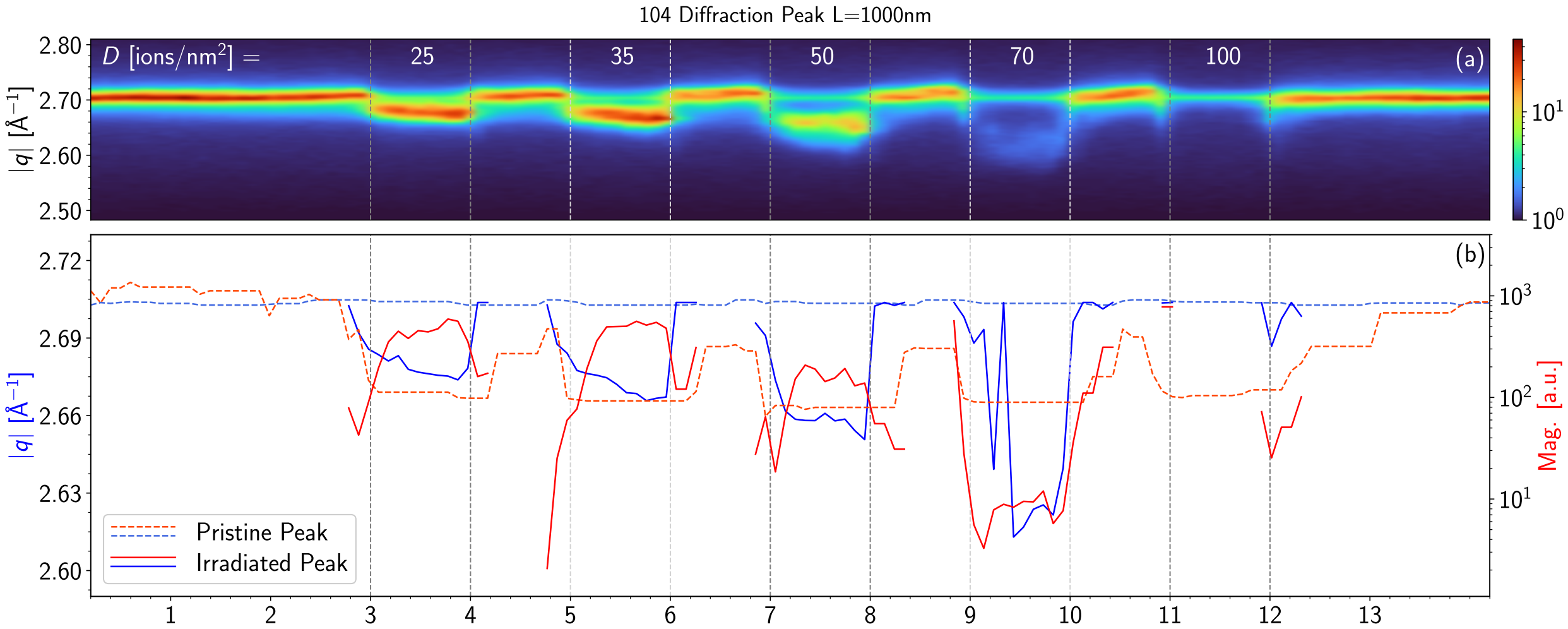

FIG. S11. (a) $|q|$-profile of the 104 peak of YBCO (integrated over q_y) at different positions across stripes of $L = 1000\,\mathrm{nm}$ with increasing dose from left to right. The dashed vertical lines denote the boundaries of irradiated regions. (b) $|q|$-positions (blue) and magnitude (red) of the Gaussian fits of the pristine (dashed lines) and diffuse (solid lines) peaks.

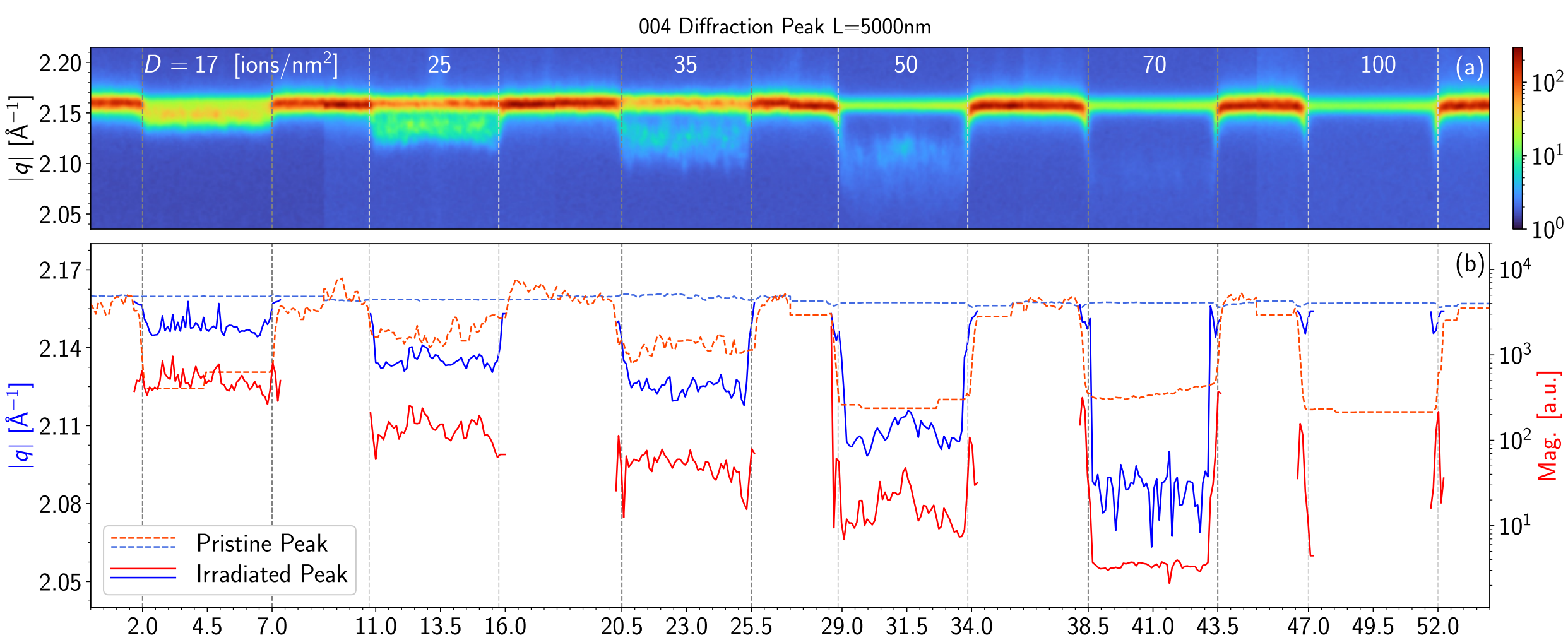

FIG. S12. (a) $|q|$-profile of the 004 peak of YBCO (integrated over q_y) at different positions across stripes of $L = 5000\,\mathrm{nm}$ with increasing dose from left to right. The dashed vertical lines denote the boundaries of irradiated regions. (b) $|q|$-positions (blue) and magnitude (red) of the Gaussian fits of the pristine (dashed lines) and diffuse (solid lines) peaks.

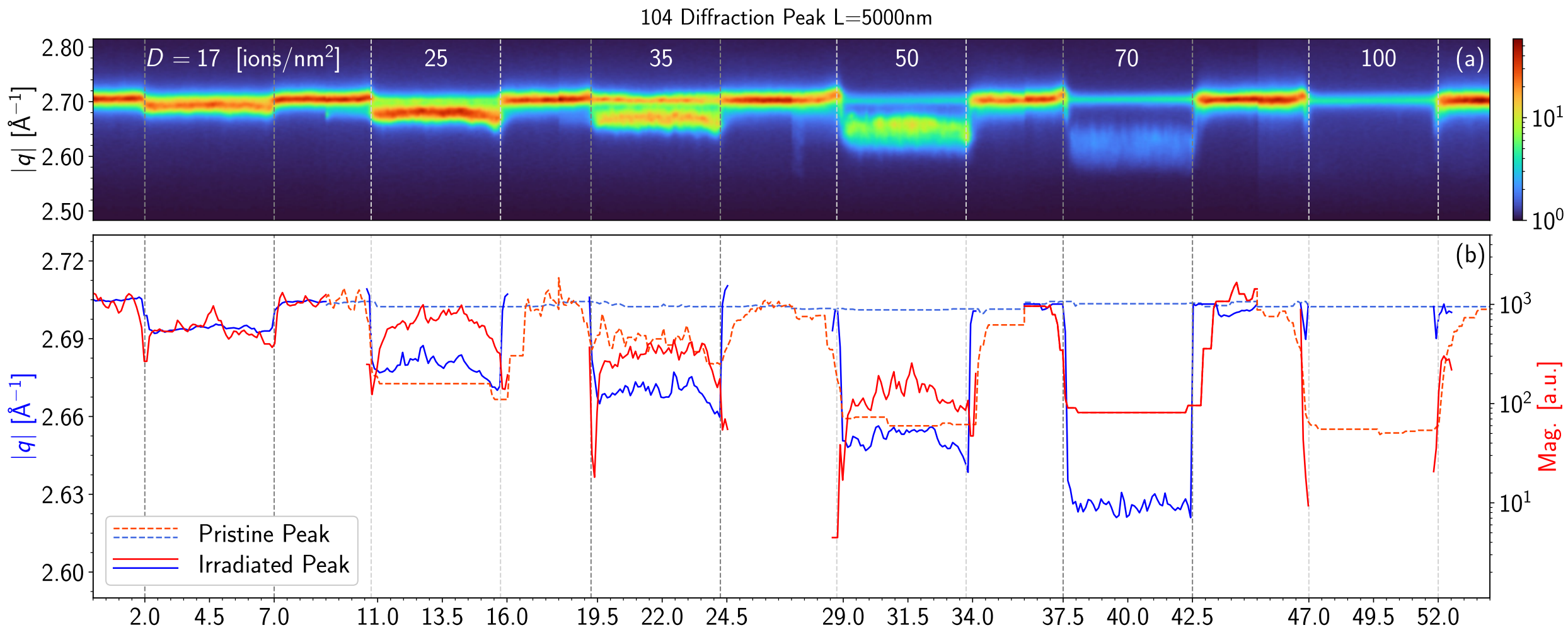

FIG. S13. (a) $|q|$-profile of the 104 peak of YBCO (integrated over q_y) at different positions across stripes of $L = 5000$ nm with increasing dose from left to right. The dashed vertical lines denote the boundaries of irradiated regions. There are outliers in magnitude for $D = 35$ ions/nm^2. Moreover, the pristine and diffuse peaks were indistinguishable from each other for $D = 17$ ions/nm^2; since there is a change in $|q|$-position, we label this single peak as a diffuse peak. (b) $|q|$-positions (blue) and magnitude (red) of the Gaussian fits of the pristine (dashed lines) and diffuse (solid lines) peaks.

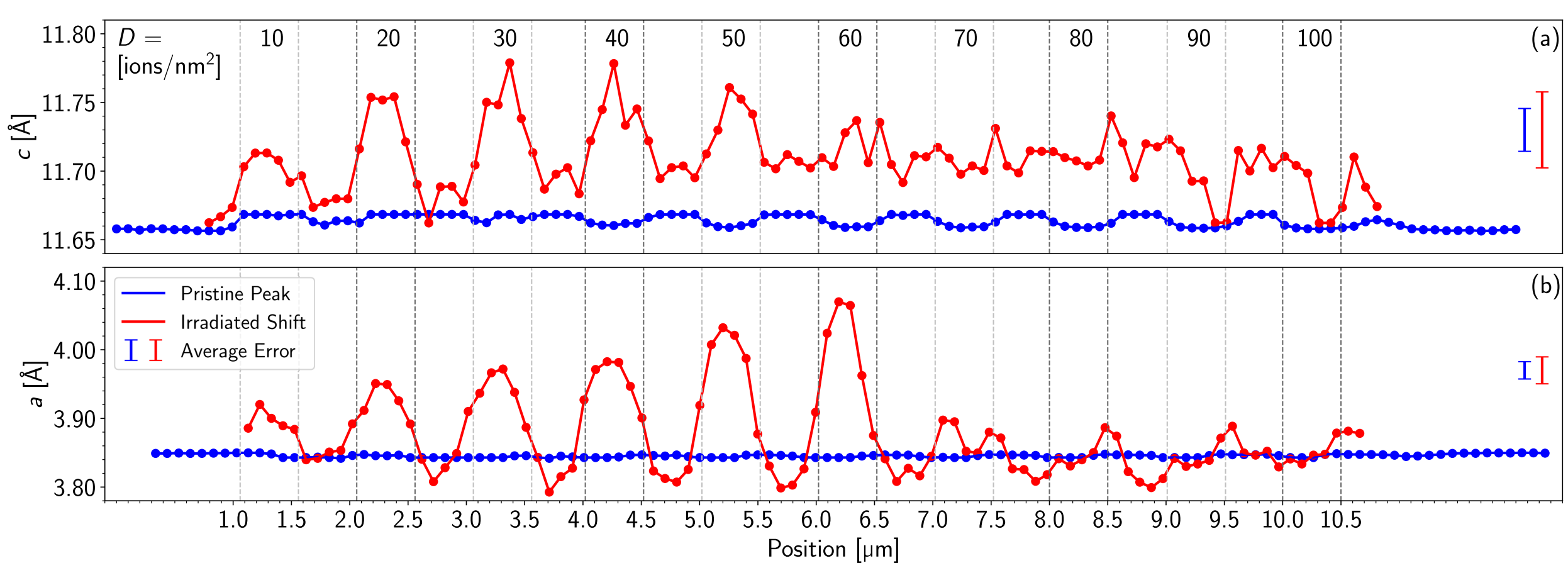

FIG. S14. Swelling of the lattice parameters c and a across YBCO film with irradiated stripes of length $L = 500$ nm. (a) Out-of-plane lattice parameter, c. (b) In-plane lattice parameter, a.

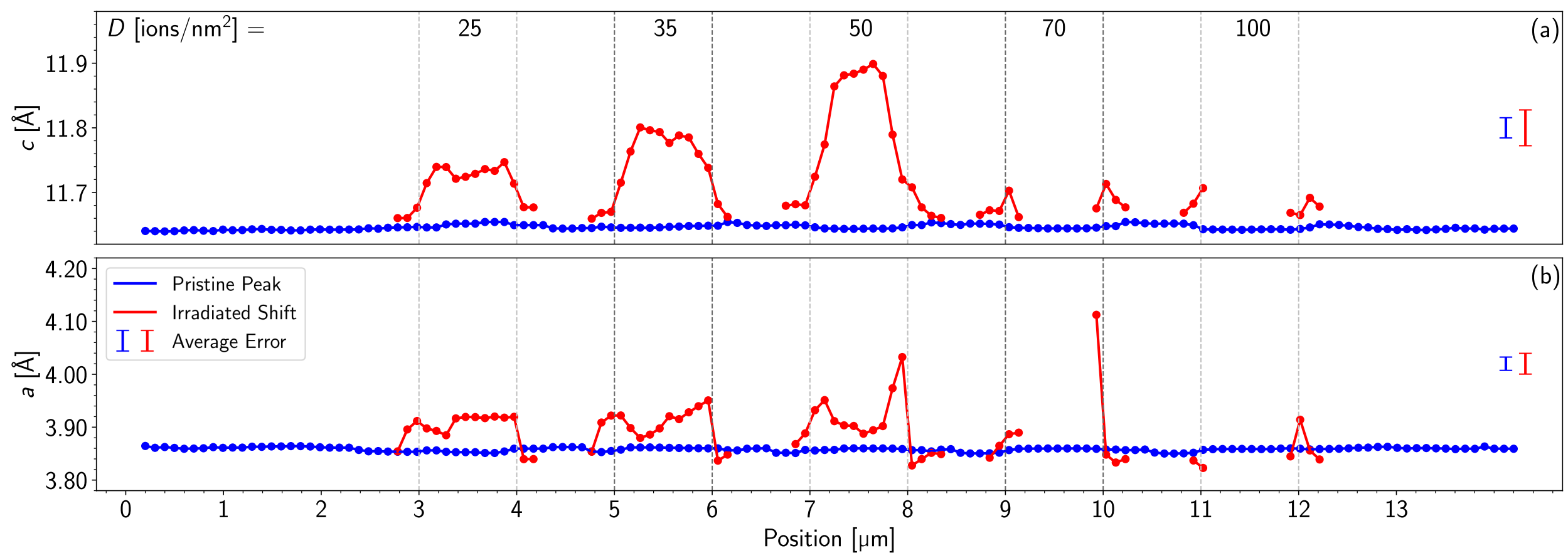

FIG. S15. Swelling of the lattice parameters c and a across YBCO film with irradiated stripes of length $L = 1000\,\text{nm}$. (a) Out-of-plane lattice parameter, c. (b) In-plane lattice parameter, a.

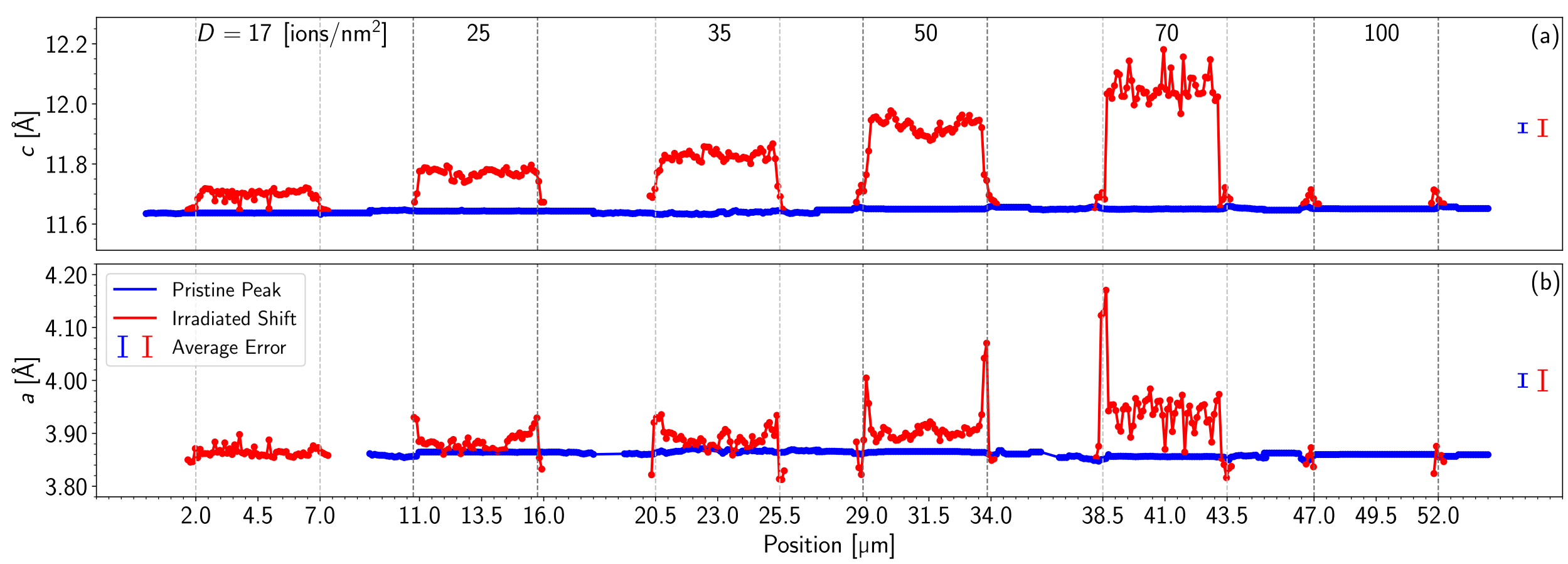

FIG. S16. Swelling of the lattice parameters c and a across YBCO film with irradiated stripes of length $L = 5000\,\text{nm}$. (a) Out-of-plane lattice parameter, c. (b) In-plane lattice parameter, a.

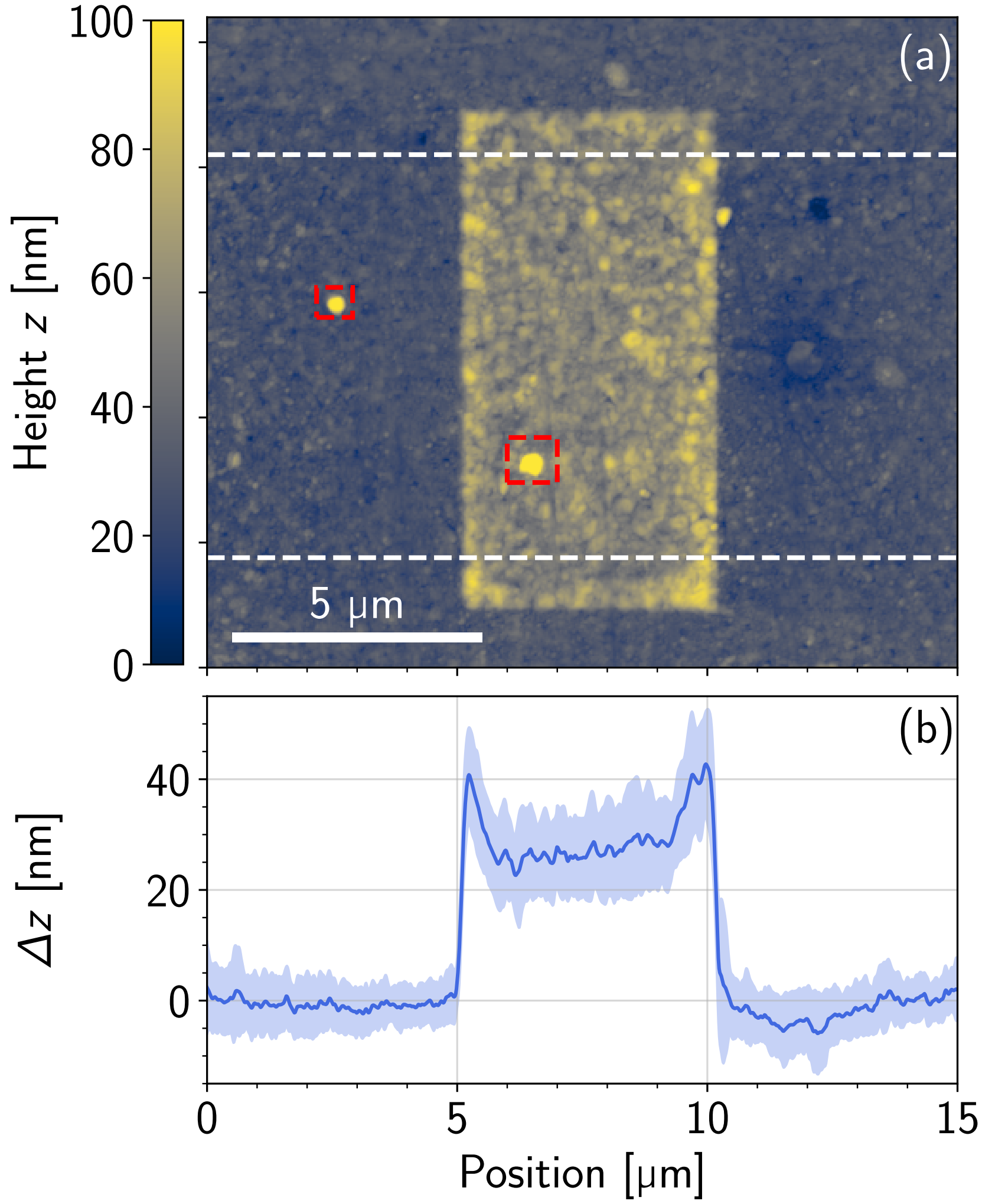

FIG. S17. (a) Exemplary AFM image (height z) measured across the area $5\,\mu\text{m} \times 10\,\mu\text{m}$ irradiated with the dose $D = 50$ ions/nm^2. The dashed, white lines represent the limits of the average profile in (b), the red boxes represent masked regions used to eliminate artefacts. (b) Averaged profile of the change in height Δz relative to the pristine YBCO region. The shaded area represents the roughness of the film.

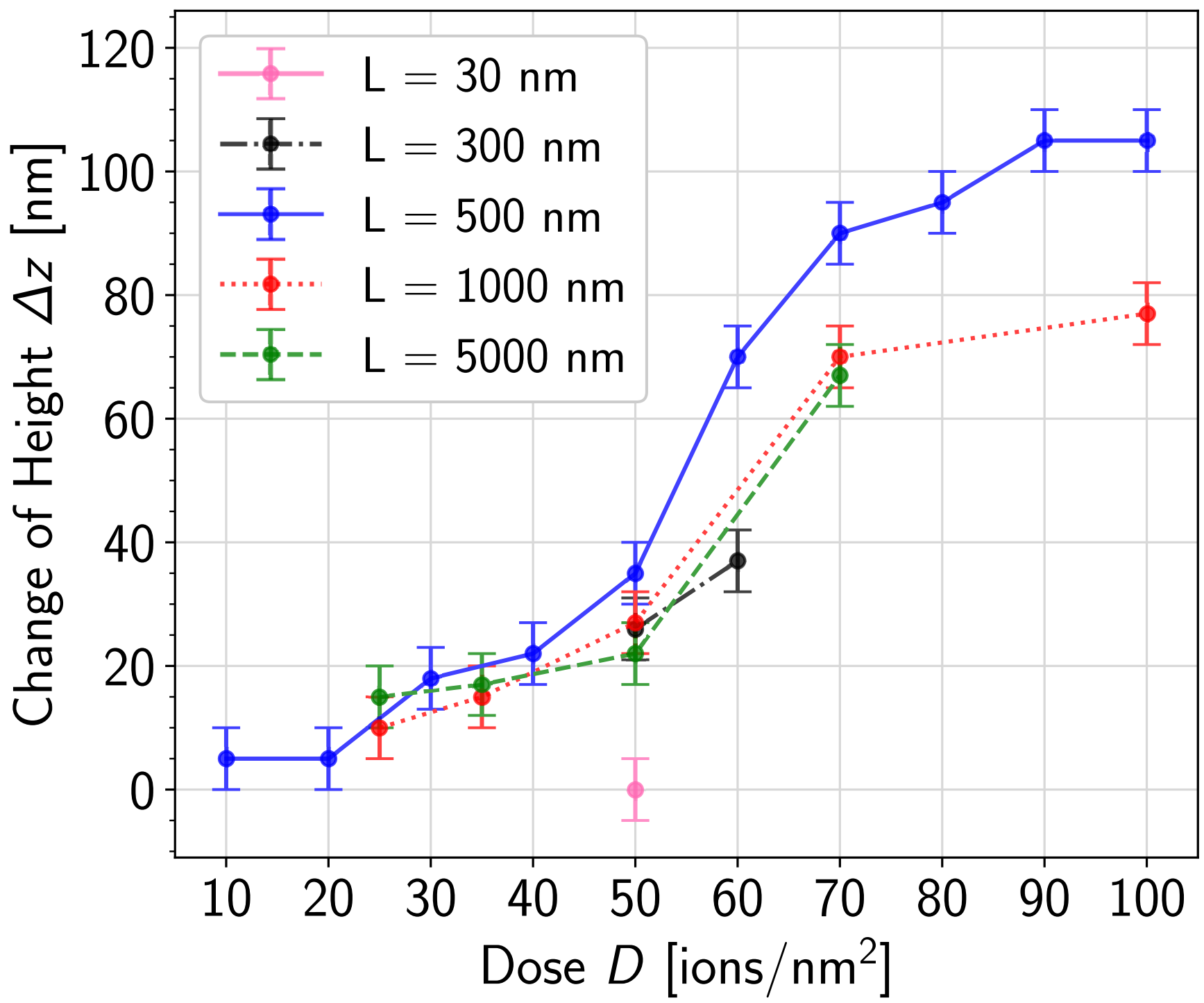

FIG. S18. Change in height Δz for each stripe length L and dose D due to bending in the substrate. Measured with AFM.
